# Automated detection of freeze-thaw signatures in archaeological sediments using deep learning

**DOI:** 10.12688/openreseurope.23697.2

**Published:** 2026-08-04

**Authors:** Sofia Kouki, Li Li, Vera Aldeias

**Affiliations:** 1 Universidade do Algarve, The Interdisciplinary Center for Archaeology and the Evolution of Human Behaviour ICArHEB, Faro, Campus de Gambelas, 8005-139, Portugal; 2 Key Reserch Base on Paleolithic Human Evolution and Paleogenetics, Institute Vetebrate Paleontology and Paleoanthropology, Chinese Academy of Science, Beijing, 100044, China; 3 Department of Human Origins, Max Planck Institute for Evolutionary Anthropology, Leipzig, 04103, Germany

**Keywords:** Micromorphology; Frost action; Deep Learning; Convolutional Neural Networks; Expert-Model collaboration

## Abstract

**Background:**

Freeze-thaw processes leave diagnostic traces in archaeological soils and sediments that are central to reconstructing past climates and understanding hominin adaptations to glacial environments. Frost features can be defined through soil micromorphology techniques using well-defined diagnostic criteria, but these traces can be subtle, variably expressed, and overlap with other pedogenic processes, making their identification time-consuming, expert-dependent, and subject to high inter-observer variability.

**Methods:**

We trained five convolutional neural network architectures on photomicrographs from eleven Plio-Pleistocene archaeological sites, implementing a two-stage classification approach by detecting first the presence of those features (binary classification) and then the feature type (multiclass classification), and validated model outputs against both interpretability analysis and a blind survey of practicing micromorphologists.

**Results:**

Results reveal a performance paradox: models achieve high performance but rely on spurious correlations rather than diagnostic criteria, while models that focus on micromorphologically relevant features show lower overall performance. Expert agreement on the same task is low, with uncertainty concentrated in feature detection rather than classification. Crucially, model and expert errors are largely independent, and each captures different aspects of frost feature recognition, establishing a basis for complementarity.

**Conclusions:**

These findings demonstrate that effective computational integration in micromorphology requires not only accurate classification but interpretability validation ensuring that model’s reason from the same diagnostic criteria as experts. We propose a human-in-the-loop approach where models provide consistent first screening, while experts offer contextual interpretation and diagnostic validation. Additionally, we present an interactive open-access tool that implements this pipeline to facilitate adoption and repeatability.

## Plain Language summary

Traces of frost action in sediments, such as distinct arrangements of soil particles and pore spaces formed by repetitive cycles of freezing and thawing, are important indicators of ancient glacial environments and how humans adapted their behavior to past cold climates. Frost features are usually identified by experts examining thin sections of soil and sediment under the microscope, a process that takes time and relies heavily on individual experience. In this study, we explore whether artificial intelligence can assist experts in recognizing frost-related features in archaeological sediments. We trained models to classify images showing different types of frost structures and compared their results with those of experienced micromorphologists who evaluated the same images. The models performed as well as human experts and sometimes recognized features that experts found difficult to classify. However, experts still provided essential insights for interpreting the meaning and context of these patterns. Together, this collaboration between human specialists and artificial intelligence offers a new way to make sediment analysis faster, more consistent, and more reproducible across archaeological research.

## Introduction

Much of our understanding of the past derives from the study of sediments and soils. The sedimentary record preserves a rich archive of information and, as such, constitutes a primary focus of research across numerous disciplines, including archaeology, soil sciences, and geology. Soil micromorphology, the microscopic study of undisturbed soil and sediment in thin sections, has become a cornerstone analytical method in these disciplines. By examining sediment composition, fabrics, pedogenic features and their subsequent micro-scale spatial relationships, micromorphologists reconstruct depositional environments, and site formation processes, revealing both natural environments and anthropogenic activities that shaped the geological and archaeological record ( Courty, 1989; Goldberg et al., 2022; Goldberg and Aldeias, 2018; Karkanas and Goldberg, 2018; Nicosia and Stoops, 2017).

Despite its central analytical power, micromorphology faces three persistent methodological constraints. First, analysis is labor-intensive and time-consuming, often requiring months of specialist effort for comprehensive site studies. Second, interpretations depend heavily on expert experience and knowledge for recognizing diagnostic features through years of training; yet these features are often subtle, variably expressed, or context dependent. Third, this interpretive complexity creates persistent inter-observer variability, reducing reproducibility, and limiting our ability to build large, comparable datasets across sites and regions ( Shahack-Gross, 2016). These challenges hinder cumulative comparisons, as quantitative syntheses are essential for reconstructing past environments and behaviors at regional scales.

One prominent example of these challenges is frost action, where repeated freeze-thaw cycles create diagnostic microscopic signatures, including granular, blocky, and platy-lenticular microstructures, capped grains, and characteristic porosity patterns ( Van Vliet-Lanoë, 1998, 1985; Vliet-Lanoë, 2010). From an archaeological perspective, recognizing these frost signatures is critical for multiple reasons. First, they enable reconstruction of past climatic conditions and understanding of hominin adaptations to glacial and periglacial settings ( Courty and Vallverdu, 2001; Davies et al., 2003; Dibble et al., 2017; Holcomb et al., 2023; Kehl et al., 2025; Murphree et al., 2025; Straus, 1991; Vachula et al., 2019; Van Vliet-Lanoë, 1998). Second, frost action affects the preservation of archaeological materials ( Deeben et al., 2010; Texier et al., 1998; Todisco and Monchot, 2008) and disturbs the spatial integrity of previously deposited archaeological assemblages ( Bertran et al., 2015, 2010; Masson, 2010).

Yet despite extensive characterization in archaeological contexts, frost features vary along a continuum from weakly developed to well-defined forms, their expression is dependent on textural features (e.g., grain-size), and they can be obliterated by or overlap with other pedogenic processes ( Bertran et al., 2017, 2015; Phillips et al., 2013; Van Vliet-Lanoë, 1998, 1985; Van Vliet-Lanoë et al., 2004). These characteristics (variability along a continuum, textural dependency, and overlap with other processes) create the conditions where expert judgment becomes inconsistent and reproducibility suffers.

These challenges are well suited to image classification. Frost feature identification is a visual pattern recognition task operating on spatially defined features (aggregate geometry, porosity, and microstructure arrangement) across large sets of images. The features themselves, while subtle and variably expressed, are diagnostically grounded in established micromorphological criteria, providing a structured classification framework. At the same time, inter-observer variability documented above indicates that consistent, automated screening could meaningfully complement expert interpretation by providing a reproducible first-pass assessment across datasets that are too large for manual analysis. Indeed, computational approaches have been adopted in related fields to address similar interpretive challenges arising from subtle features, contextual variability, and observer subjectivity. In geological petrography, where similar interpretive challenges exist, Convolutional Neural Networks (CNNs) approaches have proven effective for diverse classification tasks, such as rock and mineral classification ( Basso et al., 2025; Dalhat and Osman, 2025; Faria et al., 2025; Iyas et al., 2020; Maitre et al., 2019; Polat et al., 2021; Su et al., 2020), microfacies classification ( Pires de Lima et al., 2020), fossil classification ( Liu et al., 2023; I. C. Romero et al., 2020a), porosity and grain size analysis ( Rubo et al., 2019; Tang et al., 2020).

However, CNNs are often treated as “black boxes”, limiting their adoption in domains where diagnostic criteria are well established and where high accuracy does not necessarily indicate that models rely on meaningful features ( Ebert-Uphoff and Hilburn, 2020). To address this, a range of interpretability methods have been developed to visualize the spatially discriminative regions that influence model predictions, including Class Activation Maps (CAMs; Barredo Arrieta et al., 2020; McGovern et al., 2019; Şahin et al., 2025) and perturbation-based approaches such as Shapley Additive Explanations (SHAP; Lundberg and Lee, 2017; Stadlhofer and Mezhuyev, 2023). These methods improve the justifiability and trustworthiness of computational approaches, making model decisions more transparent and interpretable ( Bergen et al., 2019; Jiang et al., 2024; Reichstein et al., 2019). Several studies in geological petrography have incorporated interpretability analysis on their classification scheme, demonstrating both the utility and challenges of interpretability analysis ( Bai et al., 2025; Seo et al., 2022; Zheng et al., 2024).

Despite these advances in geological petrography and the shared interpretative challenges, the application of machine learning to archaeological micromorphology remains remarkably limited. To date, Arnay et al. (2026, 2021) have demonstrated the viability of deep learning for archaeological thin section analysis, first for pore type classification and more recently for segmentation of bones, charcoals, and lithic materials and estimate their subsequent abundance in thin sections. Taking a distinct methodological approach, Zickel et al. (2024) developed a GIS-based toolbox that applies semi-supervised random forest classification, rather than deep learning, to multi-modal imagery in a multi-band raster for pixel-level classification and spatial quantification of sediment components. To our knowledge, no studies have yet addressed frost feature identification in archaeological sediments, compared models and expert performance on micromorphological classification tasks or used interpretability analysis to evaluate whether models learn micromorphologically meaningful features.

Here, we address this gap by evaluating machine learning and expert performance in parallel to the classification of frost-related features in archaeological thin sections. First, we develop and evaluate CNN-based models that identify and classify frost features in photomicrographs. Second, we apply interpretability analysis to visualize model attention patterns and assess whether models learn micromorphologically meaningful diagnostic criteria. Third, we quantify inter-expert variability in frost classification, establishing a baseline for practicing micromorphologists’ performance. Fourth, we directly compare model and expert classifications to evaluate whether computational approaches provide complementary capabilities. Together, these analyses provide a framework for assessing how automated approaches can meaningfully support identification and classification of frost features in archaeological micromorphology.

## Materials and methods

### Site selection and data preparation

**Figure 1.**
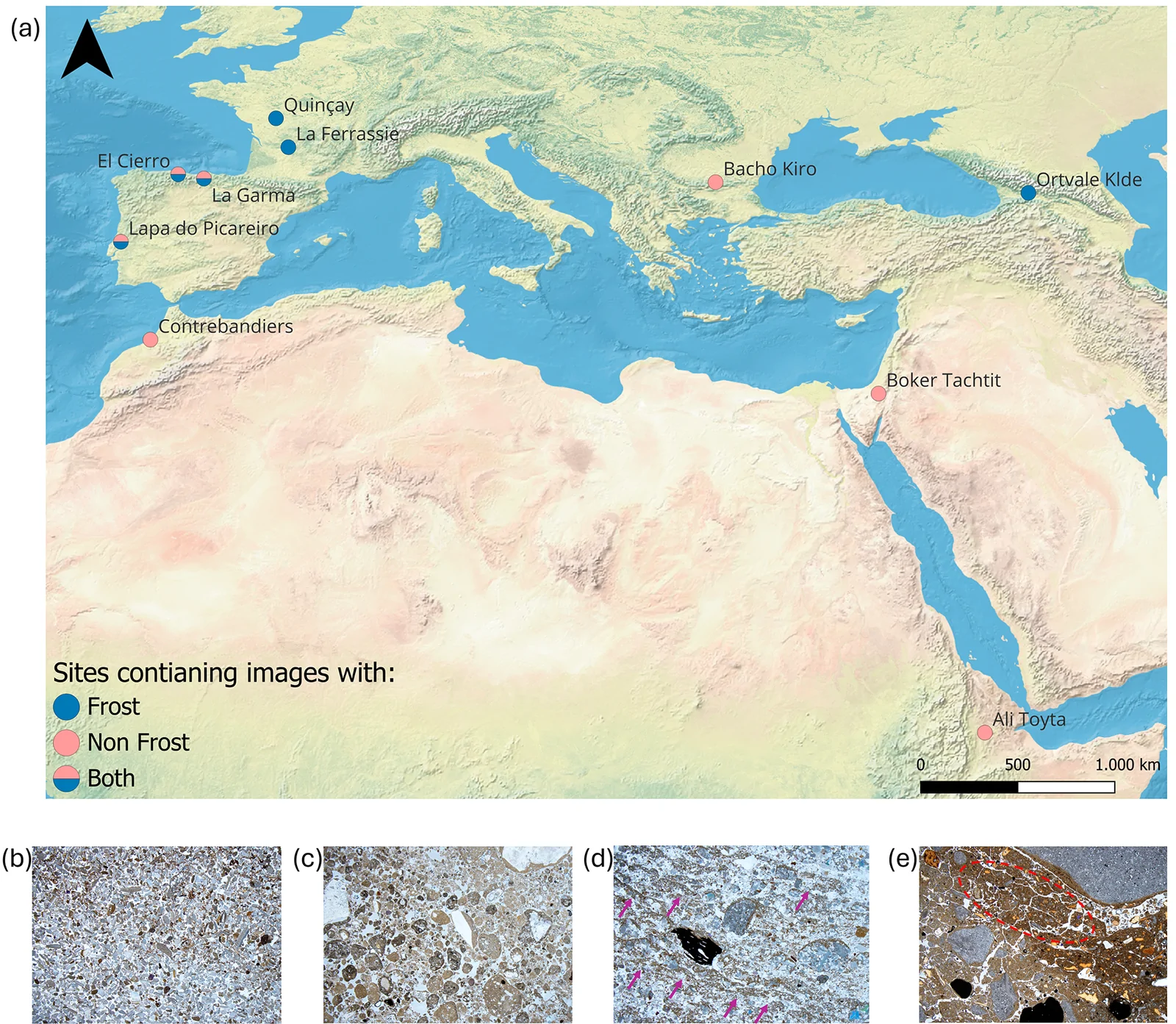
Geographic distribution of the sites and representative sample photomicrographs. (a) Map showing the sites used for frost feature identification and classification. Blue cycles indicate sites where images contain frost features (frost, and subsequent microstructure types: granular, blocky, platy-lenticular). Pink cycles indicate sites where images contain no frost features. Half-pink, half-blue cycles indicate mixed sites containing layers with either frost and non-frost features. Digital Elevation Model (DEM) data sourced from Natural Earth (public domain; https://www.naturalearthdata.com/). Map created using QGIS 3.40.6. (b-d) Thin-section photomicrographs. (b) Photomicrograph with non-frost features from Contrebandiers site showing geogenic bioclastic sands ( Aldeias et al., 2014). (c) Photomicrograph from Quinçay showing frost features with granular microstructure. (d) Photomicrograph from La Ferrassie showing frost features with platy-lenticular microstructures (banded fabric; purple arrows) ( Aldeias et al., 2023). (e) Photomicrograph from Ortvale Klde showing frost features with blocky microstructures. The dashed red ellipse shows sub-angular blocky microstructure. An interactive map showing representative photomicrographs from all sites is provided in data repository ( 10.17605/OSF.IO/TYDSM).

We selected thin section samples from Plio-Pleistocene archaeological sites spanning a broad range of geographical and environmental contexts, specifically targeting deposits containing frost-affected and non-frost-affected sediments. European sites included Quinçay and La Ferrassie (France), Ortvale Klde (Republic of Georgia), El Cierro, La Garma (Spain), Lapa do Picareiro (Portugal), and Bacho Kiro (Bulgaria). Sites beyond Europe included Boker Tachtit (Israel), Ali Toyta (Ethiopia), and Contrebandiers Cave (Morocco). We chose these sites because they represent different periglacial and non-periglacial depositional environments with the hope to build a model that can (later) be generalized across diverse archaeological settings ( Figure 1).

We analyzed 154 thin sections, yielding 733 photomicrographs captured under plane-polarized light (PPL) using a Nikon SZM25 stereomicroscope equipped with a DS-Fi3 digital camera at 5x magnification and 1x zoom. At lower magnifications (e.g., scanned thin section images) the broader depositional context is visible, but individual diagnostic features, such as aggregate boundaries and shape, porosity distribution, lack sufficient resolution for the classification. At higher magnifications, fine details are resolved, but the spatial relationships between features that define the microstructure (granular, blocky, platy-lenticular) types fall outside the field of view. In our case, a 5x magnification provides a balance between the feature resolution and spatial context, which is necessary for the classification. We also used PPL entirely to create a controlled, single-modality imaging procedure, which is particularly useful since diagnostic criteria are directly observed under PPL. Also, merging PPL and XPL computationally would necessitate either multi-model fusion structures lacking proven methods in micromorphological imaging or the possibility of adding methodological artifacts through correlated duplicate observations in the same region.

All the images we acquired are in RGB format, with a of 2880 x 2048-pixel and saved as uncompressed .tif files. These raw images formed the input dataset for subsequent data preparation and model training described in Section 2.2.

We cropped each photomicrograph into six equal parts to capture local textural variability, and we excluded areas with no relevant sedimentary information (e.g., slide edges, large rock fragments). This allowed us to increase dataset size and ensure models learn from localized diagnostic features suitable for classification ( Lakshmanan et al., 2021). After cropping, we resized the images to 512 x 512 (width x height) pixels, ensuring that all images are the same size before model implementation.

We established reference labels based on two annotation levels: (1) binary classification (Frost vs. non-frost), and (2) multiclass classification of Frost images into Blocky, Granular, or Platy-lenticular microstructure types following the standardized micromorphological nomenclature ( Stoops, 2021), though simplified here to enhance the practical applicability.

Our dataset consist of 921 images in total and randomly split into training (70%), validation (15%) and test (15%) sets (Supplementary Information 4, Table S4.2.1). We augmented only the training set with random rotations, flips, and height/width shifts. This allows us to increase the training dataset images to avoid overfitting. Since some image classes contain more images than others, and prevent overconfident predictions on minority classes, we addressed this class imbalance by computing class weights and applying label smoothing ( Müller et al., 2019; Spelmen and Porkodi, 2018).

### Machine Learning workflow

We applied five CNN architectures for the two-stage classification framework: MobileNetV2, EfficientNetV2B0, ResNet50, a custom CNN, and a custom Bayesian Neural Network (BNN). On one hand, we selected three widely adopted CNN initialized with ImageNet weights models (MobileNetV2, EfficientNetV2B0, ResNet50) to leverage transfer learning, which provides low-level feature extraction (such as edges and textures) on small-to-medium datasets without requiring massive training volumes dataset ( Lakshmanan et al., 2021). Specifically, MobileNetV2 emphasizes computational speed and has a simple feature extraction design by using inverted residuals ( Sandler et al., 2019). ResNet50 uses a more complex layered structure with shortcut mappings to capture detailed patterns and prevent the vanishing gradient problem ( He et al., 2015), while EfficientNetV2B0 provides a balanced approach by combining both computational efficiency and complex detection capability via optimized compound scaling ( Tan and Le, 2021).

On the other hand, Custom CNN and BNN models were designed from scratch specifically for this task to force the networks attention onto domain-specific matrix textures rather complex, object-centric attention inherited from ImageNet pretraining. We selected BNN specifically because it allows for uncertainty estimation (confidence intervals) in predictions and to do so we used Monte Carlo (MC) dropout, executing 20 stochastic forward passes per image to quantify the epistemic uncertainty (model parameter variance) of our classifications ( Jospin et al., 2022; Wang and Yeung, 2021).

To evaluate all models’ generalization, we applied 5-fold stratified cross-validation (CV) with each fold presenting class proportions and ensuring that every image is used for validation once in one of the five subsets. We chose stratified over random cross-validation to maintain consistency across class proportions across all folds, reducing the variance in performance estimates, especially for minority classes ( Ghojogh and Crowley, 2023; Gorriz et al., 2024). After completing CV, a final model for each one of the CNN-based models was trained on the entire augmented training set and evaluated on an independent held-out test set, providing an unbiased estimate of model performance on unseen data ( Ghojogh and Crowley, 2023).

For all models, we used ReLU as the activation function to introduce non-linearity while avoiding saturation, followed by global average pooling and fully connected dense layers to reduce spatial dimensions and avoid overfitting, and fully connected dense layers to compute final class probabilities. To prevent overfitting, we applied high dropout rates (0.3–0.5) to randomly deactivate neurons during training steps, l2 regularization to penalize large weight values ( Gavrilov et al., 2018), and adaptive learning rate scheduling to systematically reduce the step size as training converged on local minima ( Santos and Papa, 2022; Ying, 2019).

We evaluated all models’ performance using accuracy, precision, recall, F1-scores, and confusion matrices, as well as visualizing the misclassified images per model ( Raschka, 2020; Tohka and van Gils, 2021). To clearly assess which model performed better and how large the performance differences were across both classification tasks, we compared metrics across all folds and the test set using the Friedman test with Nemenyi post-hoc tests to evaluate significance between models ( Demšar, 2006a; Rainio et al., 2024).

To understand which parts of an image influences the model’s decision-making process, we applied Score Class Activation Map (Score-CAM), a visualization technique that generates maps which highlight the image regions that mostly affect a model decision that an image belongs to one class or another ( Minh, 2023; Wang et al., 2020). To see if these models can be used for archaeological deployment and if the models learn meaningful patterns, we also qualitatively compared if these activation maps align with the established micromorphological criteria, such as frost-related porosity, aggregate shape and arrangement, or microstructure geometry ( Stoops, 2021; Van Vliet-Lanoë, 1985; Vliet-Lanoë, 2010). In addition to qualitative inspection, we quantified Score-CAM attention concentration by summarizing what proportion of pixels is needed to reach fixed activation mass thresholds per model and per image. Full implementation details are provided in Supplementary Information 4 (Section S4.2.2).

We determined optimal hyperparameter values for learning rates, dropout rates, regularization coefficients, and models’ architecture-specific parameters (e.g., number of dense layers) through systematic iterative experimentations. We tested variations of these hyperparameter values, evaluated performance on the validation set, and refined settings based on results. This process balanced multiple objectives: maximized accuracy while preventing overfitting and ensuring stable convergence. The final hyperparameter configurations for all models are reported in Supplementary Information 4. Table S4.2.2.

Since the random splitting operates at crop level, some crops form the same photomicrograph may appear in different sets. However, in our case, the photomicrograph-level split was not feasible because it would have drastically reduced sample size given the already limited dataset. To assess whether the crop-level splitting substantially inflates performance estimates, we performed a small robustness test on full photomicrographs (n = 21; 11 frost, 10 non-frost), applying all five models and computing the same metrics used for the cropped images, together with Score-CAM maps to assess the whole-image behavior (See Supplementary Information 4, Section S4.3 for details).

### Expert survey design

Model performance metrics alone cannot assess whether experts would agree with model classifications or whether the reference labels themselves are reliable. Therefore, we conducted an anonymous online survey among practicing micromorphologists (Microsoft Forms v25.7), distributed via direct invitations to micromorphologists and remained open from May 10, 2025, to October 31, 2025.

The survey serves three purposes: (1) quantify inter-expert agreement to identify potential label noise in the training data, (2) establish whether certain images are inherently ambiguous even to experts, and (3) provide a baseline for further comparison between CNN performance to human expert performance.

We selected a subset of 23 images that are representative of our dataset in a two-stage process. First, we identified potentially ambiguous images based on frequent misclassification by preliminary CNN models, (misclassified by ≥3 of the models), and cases where reference labels require extended expert discussion without reaching initial consensus. Second, we refined this subset through expert review to ensure representation of specific ambiguity types, such as poorly defined frost features, frost features not dominating the field of view, and non-frost images displaying potentially confounding characteristics.

In the first task (first screening assessment, n = 9), participants evaluated whether the sediment features within the resized image are sufficient to attempt classification. We selected these 9 images specifically because preliminary CNN models frequently misclassified them and because reference label assignment required extended discussion, suggesting that diagnostic features were difficult to identify at the cropped and resized scale.

In the second task (binary and multiclass classification, n = 14), participants classified images in a two-stage process mirroring the CNN framework: first assessing whether images contain any frost features, and if they do, then the specific type of microstructure. This conditional design prevents forced classification and allows direct comparison with model outputs.

We assessed inter-rater agreement using Fleiss’ kappa, individual expert-reference labels alignment using Cohen’s kappa ( Landis and Koch, 1977), raw percentage agreement, and relationships between response variability and agreement using Spearman’s ρ ( Mukaka, 2012). We also quantified the proportion of “Unsure” responses per participant and per image. Full statistical procedures are detailed in Supplementary Information 4, Section S4.4.

### Experts and CNN models alignment

To evaluate whether CNN-based models align with expert micromorphological practice, we compared model predictions and expert classifications across the 14 images from the survey’s second tasks, the images where both experts and models completed full two-stage classification. All comparisons used the reference labels as reference.

Evaluating experts and CNN models’ alignment addresses three questions: (1) do CNN models agree with expert classifications, (2) do they make similar errors on the same images, and (3) does model uncertainty correspond with expert-perceived classification difficulty.

To evaluate how models behave on images where experts judged diagnostic features as insufficient for classification, we used the nine images from the survey’s first-screening task. Since these nine images presented challenges in feature visibility at the cropped and resized scale, they were exclusively for assessing whether models can distinguish between images with sufficient and insufficient diagnostic information. The remaining 14 images, where features were considered sufficiently visible for classification, were used for the two-stage classification tasks (binary and multiclass) and form the basis of the expert-model comparison described below. For the nine first-screening images, we categorized each by majority expert vote as “Visible”, “Not visible”, or “Unsure”. For each model, we generated frost/non-frost prediction on all nine images and summarized (i) class proportions on “Not visible” images, (ii) mean confidence scores (Equation 1, Supplementary Information 4, Section 4.5) for “Visible” vs. “Not visible’ subsets, and (iii) For the BNN, Monte Carlo dropout uncertainty (standard deviation over 20 passes).

For the classification task, we assessed the overall degree of agreement using Fleiss’ kappa first for the CNN models only (n = 5) and then all participants combined (n = 24; 5 models and 19 experts). We also computed Cohen’s kappa for all 95 expert-model pairs and classification accuracy against reference labels for each participant. To test whether experts agreed more with one another than with models, we compared Cohen’s kappa distributions using Mann-Whitney U test ( Conover, 1999), with the same comparison applied to accuracy values. We quantified the error overlap between experts and models using Spearman’s ρ between misclassification patterns. We also used Spearman’s ρ to examine relationships between agreement proportions, model confidence scores, and BNN uncertainty metrics.

We categorized images into difficulty categories based on classification performance: (1) “Hard for CNN” correctly classified by ≥70% of experts but misclassified by ≥3 models; (2) “Hard for Experts” correctly classified by ≥3 models but correctly classified by <70% of experts; (3) “Hard for Both” misclassified by ≥3 models and correctly classified by <70% of experts. These thresholds were selected to identify images with clear difficulty patterns while maintaining sufficient sample sizes per category. Complete statistical pipelines and details are provided in Supplementary Information 4.

### Workflow and interactive classification tool

The entire workflow, including image pre-processing, classification, evaluation, and interpretability were implemented in Python 3.12 with Tensorflow 2.20.00 ( Abadi et al., 2016) and Keras 3.12.2 ( Chollet, 2015) libraries within Google Colaboratory ( Bisong, 2019) using an A100 GPU. For the three off-the-shelf models, ImageNet pretrained weights were loaded directly through Keras API (MobileNetV2: Sandler et al., 2019; EfficientNetV2B0: Tan and Le, 2021; ResNet50: He et al., 2015).

**Figure 2.**
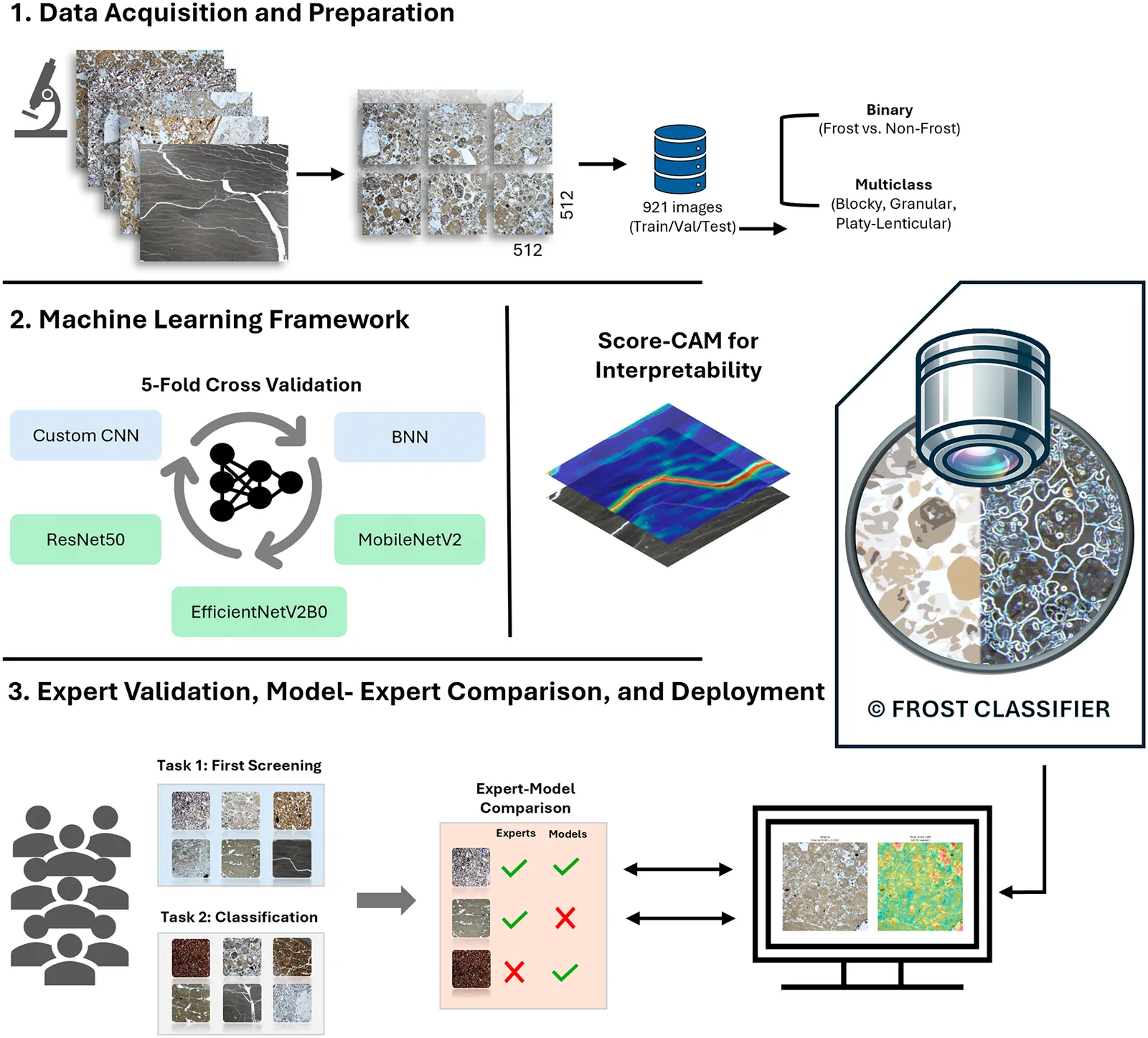
Frost classification workflow. End-to-end methodology, starting from the data acquisition and acquisition, model training and validation, expert survey and comparison with model outputs, and final deployment of the classifier as interactive tool.

Statistical analyses of the survey data and model comparisons were performed using Scikit-learn ( Pedregosa et al., 2011) and Statsmodels ( Seabold and Perktold, 2010). Our workflow is presented in Figure 2 and a detailed glossary of the machine learning and statistical terms used in this study is included in Supplementary Information 4, Table S4.1.1.

To support practical adoption and reproducibility, we also developed an interactive web application using Gradio ( Abid et al., 2019) and Hugging Faces that enables users to upload their thin section photomicrographs and obtain frost classification predictions with confidence scores, uncertainty and interpretability, implementing the same two-stage classification framework (binary followed by multiclass). The tool is publicly available at online ( https://huggingface.co/spaces/skkou/frost_classifier) and detailed guidelines on how to use it are provided in Supplementary material 4, Section S4.8.

## Results

### CNN models achieve high performance but rely on spurious correlations

Overall, all five models (MobileNetV2, EfficientNetV2B0, ResNet50, Custom CNN, and BNN) achieve high classification performance both binary (frost vs. non-frost) and multiclass (blocky, granular, and platy-lenticular) tasks in both 5-fold cross-validation and the test set, with accuracy ranging from 94-99% in binary and 86-96% in multiclass. Models achieve better performance on binary tasks, while distinguishing among the three frost-related microstructures is more challenging (see Supplementary Information 1 and 4; Section S4.6–7).

**Figure 3.**
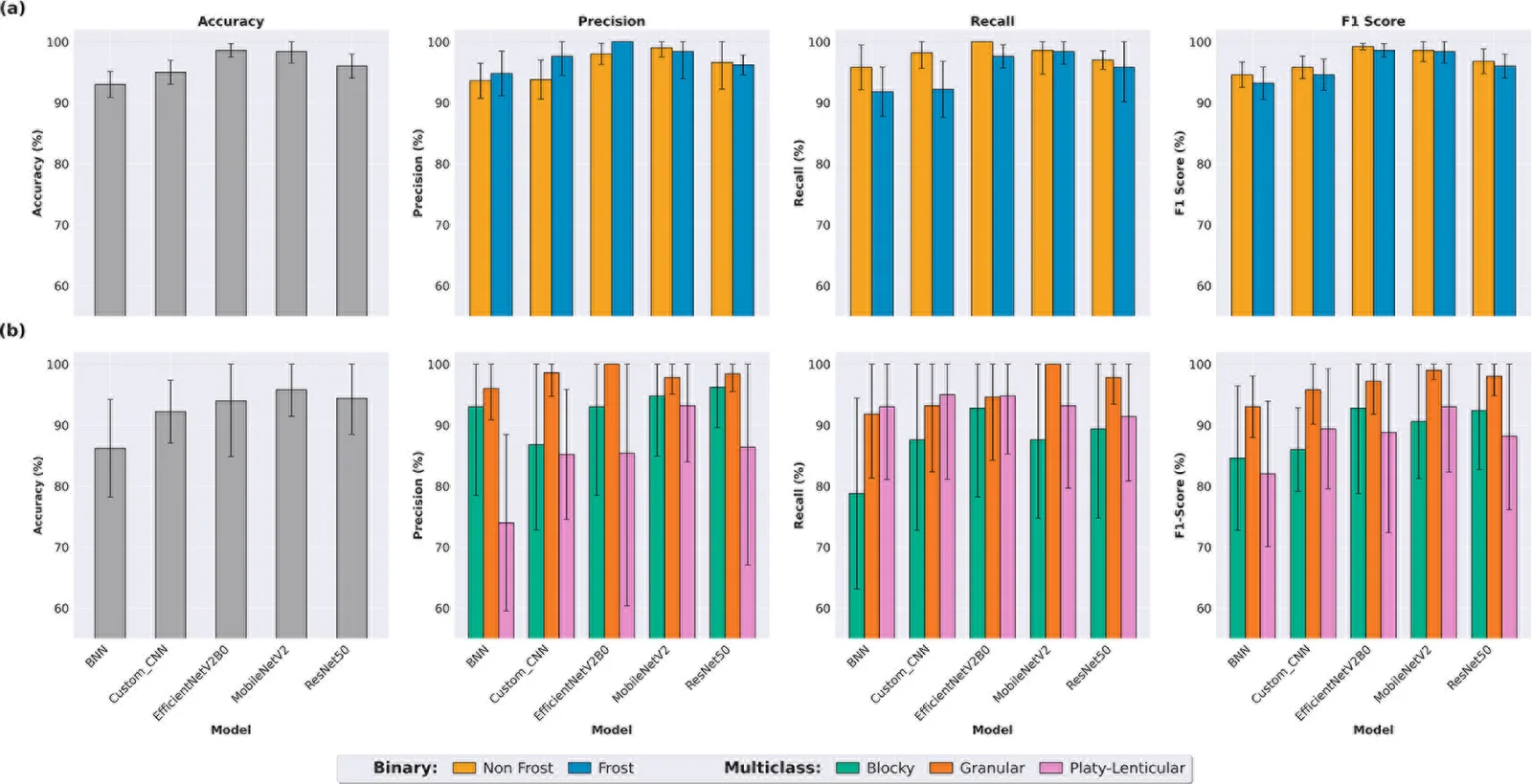
Comparative performance of five CNN architectures (BNN, Custom CNN, EfficientNetV2b0, MobileNetV2, and ResNet50) based on mean Accuracy, and per class Precision, Recall, and F1-Score and the standard deviation over 5-Fold cross-validation (CV). (a) Binary classification (Frost vs. non-frost) performance comparison. (b) Multiclass classification performance across Blocky, Granular, and Platy-lenticular microstructure classes. Error bars represent the 95% confidence intervals.

Learning dynamics shows stable convergence for all models in the binary task, while there are more variable validation patterns with oscillations during the training procedure in the multiclass task (Supplementary Information 4, Figure S4.6.1-S4.6.3). Cross-validation results (precision, recall, and F1-score) are summarized in Figure 3. In binary classification, MobileNetV2 and EfficientNetV2B0 achieve the highest mean accuracy (98–99%), with all other models above 94%. In multiclass classification, performance is slightly lower. MobileNetV2 performs best (~ 96%), followed by EfficientNetV2B0 and ResNet50 (~ 94–95%). Images with granular features are classified consistently across all models (>93% precision and recall), while images with blocky and platy-lenticular features show greater variability. Evaluation of the test set confirms these cross-validation patterns, where MobileNetV2 and EfficientNetV2B0 achieve the highest performance on binary classification and show slightly lower performance in multiclass classification (Supplementary Information, Tables S4.6.1–2). Statistical comparisons using Friedman’s test confirm significant differences in binary classification, with MobileNetV2 and EfficientNetV2B0 achieving the highest ranks. No significant differences are detected in the multiclass classification ( Table 1A).

**Table 1.** Statistical comparison of CNN model performance and expert-model agreement across 14 classification images. (A) Model ranking based on Friedman’s test with significant pairwise differences identified by Nemenyi post-hoc test. (B) Pairwise agreement comparison between expert-model pari and expert-expert pairs, assessed using Mann-Whitney U test. (C) Group-level agreement using Fleiss’ kappa in three configurations (inter-expert, inter-model, and models-experts).

A. Model ranking and significant pairwise differences		
Model	Rank	Significant differences
MobileNetV2	1	>BNN (p = 0.039); >ResNet50 (p = 0.001)
EfficientNetV2B0	2	>ResNet50 (p = 0.005)
Custom CNN	3	-
BNN	4	-
ResNet50	5	-

B. Expert-model vs expert-expert pairwise agreement				
Comparison	Cohen’s kappa (SD)	Accuracy (SD)	N pairs	Statistical test
Expert-Model	0.387 (0.343)	70.4 (21.1)	95	U = 7397, p = 0.224
Expert-Expert	0.447 (0.361)	74.1 (21.2)	171	Reference
Δ (difference)	−0.060	−3.7	-	Not significant

C. Group agreement configurations				
Configuration	Fleiss’ kappa	Mean agreement (%)	N raters	Interpretation
Inter-Expert	0.202	60.9	19	Slight
Inter-Model	0.676	91.4	5	Substantial
Models-Experts	0.212	64.6	24	Fair

Note: Friedman x2 = 33.4, p = < 0.0001. Nemenyi post-hoc test, a = 0.05.

**Figure 4.**
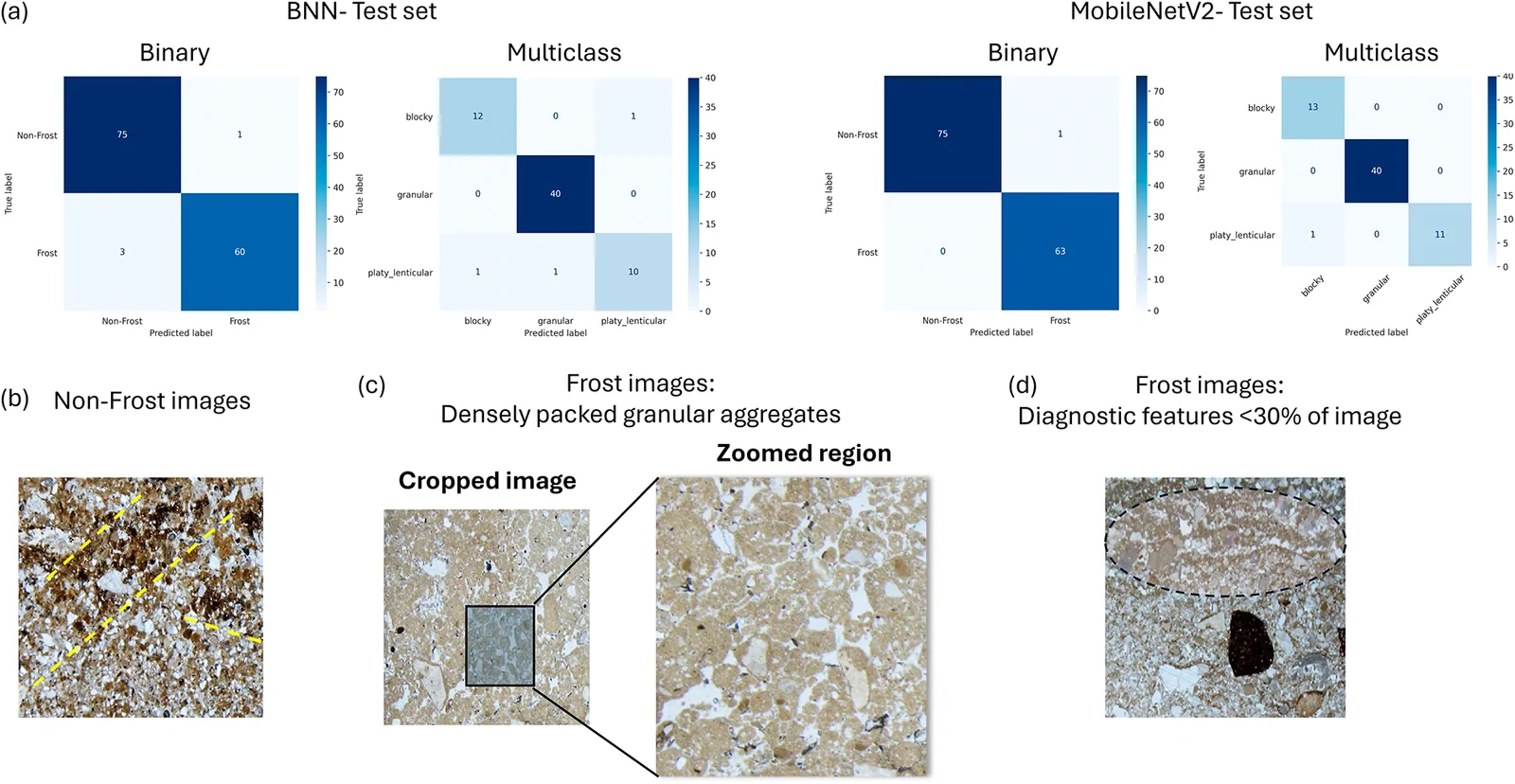
Systematic misclassification patterns across CNN models and representative examples of challenging images. (a) Confusion matrices on the test set for the best-performing off-the-shelf model (MobileNetV2) and a custom model (BNN) for binary (Frost vs. Non-frost) and multiclass (granular, blocky, platy-lenticular) classification tasks. (b) Representative Non-Frost images exhibiting high spatial heterogeneity. The complex mixed matrix with no dominant textural patterns mimics frost patterns (areas between the dashed yellow lines). (c) Representative frost images with densely packed granular aggregates where frost-related pore space (white) is obscured by tightly packed aggregates, as shown in the zoomed inset. (d) Representative frost images where diagnostic features occupy less than 30% of the image area. The dashed orange ellipse indicates the spatial extent of the diagnostic region within the cropped photomicrograph.

Misclassification patterns are consistent in both binary and multiclass classification ( Figure 4). In the binary classification, non-frost images contain high spatial heterogeneity, while in the frost class and the subsequent microstructure classification, misclassifications are concentrated in images where the diagnostic features are either poorly developed, densely packed or occupy less than 30% of the image area.

**Figure 5.**
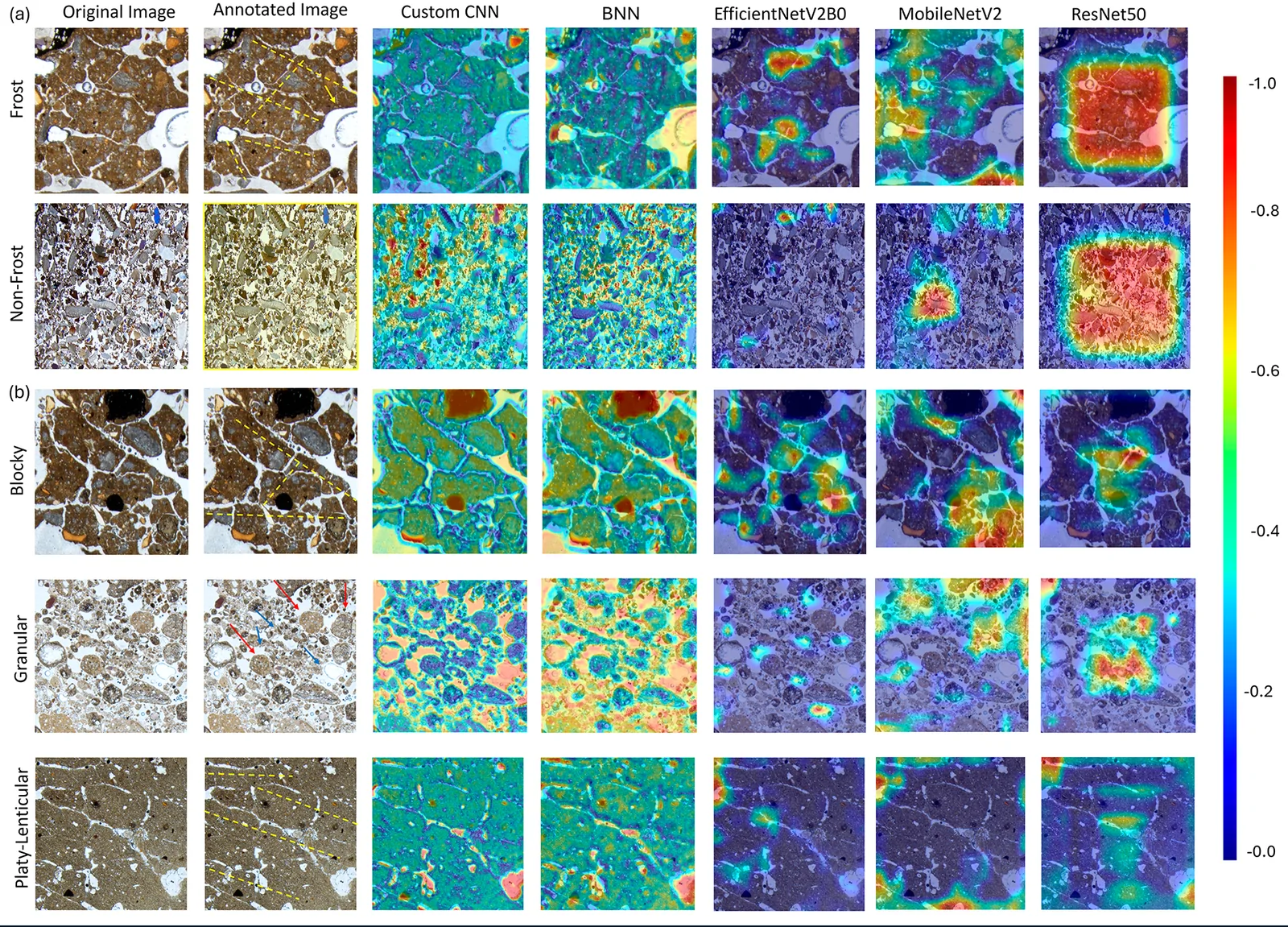
Score-CAM visualizations across five CNN architectures illustrating model attention patterns during (a) binary (Frost vs. non-frost) and (b) multiclass (Blocky vs. Granular vs. Platy-lenticular) classification. For each class, the same image is used across all five models to allow direct comparison of attention patterns. Original images are in PPL, where the porous space is white. Each heatmap highlights the image regions contributing most to model predictions. Differences in activation distribution reveal model-dependent focus and spurious correlations (for EfficientNetV2B0, MobileNetV2, and ResNet50), whereas other models (BNN and Custom CNN) emphasize more on diagnostically relevant areas (e.g., porosity) consistent with micromorphological criteria.

To assess whether models rely on micromorphologically diagnostic features (such as aggregate geometry, frost-related porosity, and sediment arrangement), we applied Score-CAM interpretability analysis. For both classification tasks, MobileNetV2, EfficientNetV2B0, and ResNet50 models focus on scattered or fixed activation patterns that do not align with diagnostic features used by micromorphologists ( Figure 5). ResNet50 consistently highlighted a central rectangular region regardless of the image content. In contrast, Custom CNN and BNN show more interpretable activation patterns (>0.6). These models show higher attention to aggregate geometry and characteristic porosity, especially in granular and platy-lenticular classes. However, both models occasionally attended to irrelevant elements within the image, such as bone fragments. The cumulative distribution of Score-CAM activation values across pixels shows that off-the-shelf models have highly concentrated attention with the top ~10–25% of pixels accounting for roughly half of the activation mass. Custom CNN and BNN require ~40–45% of pixels to reach the same threshold (Supplementary Information 4, Figure S4.6.6).

Quantitative Score-CAM analysis shows differences in attention concentration across architectures. Off-the-shelf models (MobileNetV2, EfficientNetV2B0, ResNet50) require only ~10–20% of pixels to reach 50% of activation mass, while Custom CNN and BNN needed approximately 40–45% of pixels showing more diffuse patterns (Supplementary Information 4, Figures S4.6.7–8 and Table S.4.6.1).

The complementary robustness test on the entire photomicrographs shows that off-the-shelf models, and particularly MobileNetV2, retained relatively balanced frost/non-frost performance. Whereas the custom CNN and BNN achieve lower performance metrics, but focused more consistently on micromorphological relevant features, with all models more prone to misclassify frost than non-frost images at whole scale (Supplementary Information 4, Figures S4.6.11–12 and Table S4.6.3).

### Expert agreement on frost identification and classification is low

Of the 20 practicing micromorphologists who responded to the survey call, 19 completed it, with 4 shelf-reporting little to no experience, 10 having some experience, and 5 having significant experience with frost action. Due to small and uneven sample sizes, the statistical analysis performed has a more exploratory and descriptive intention rather than a definitive and inferential one. The survey’s raw data and extended results are provided in Supplementary Information 2 and 4.

**Figure 6.**
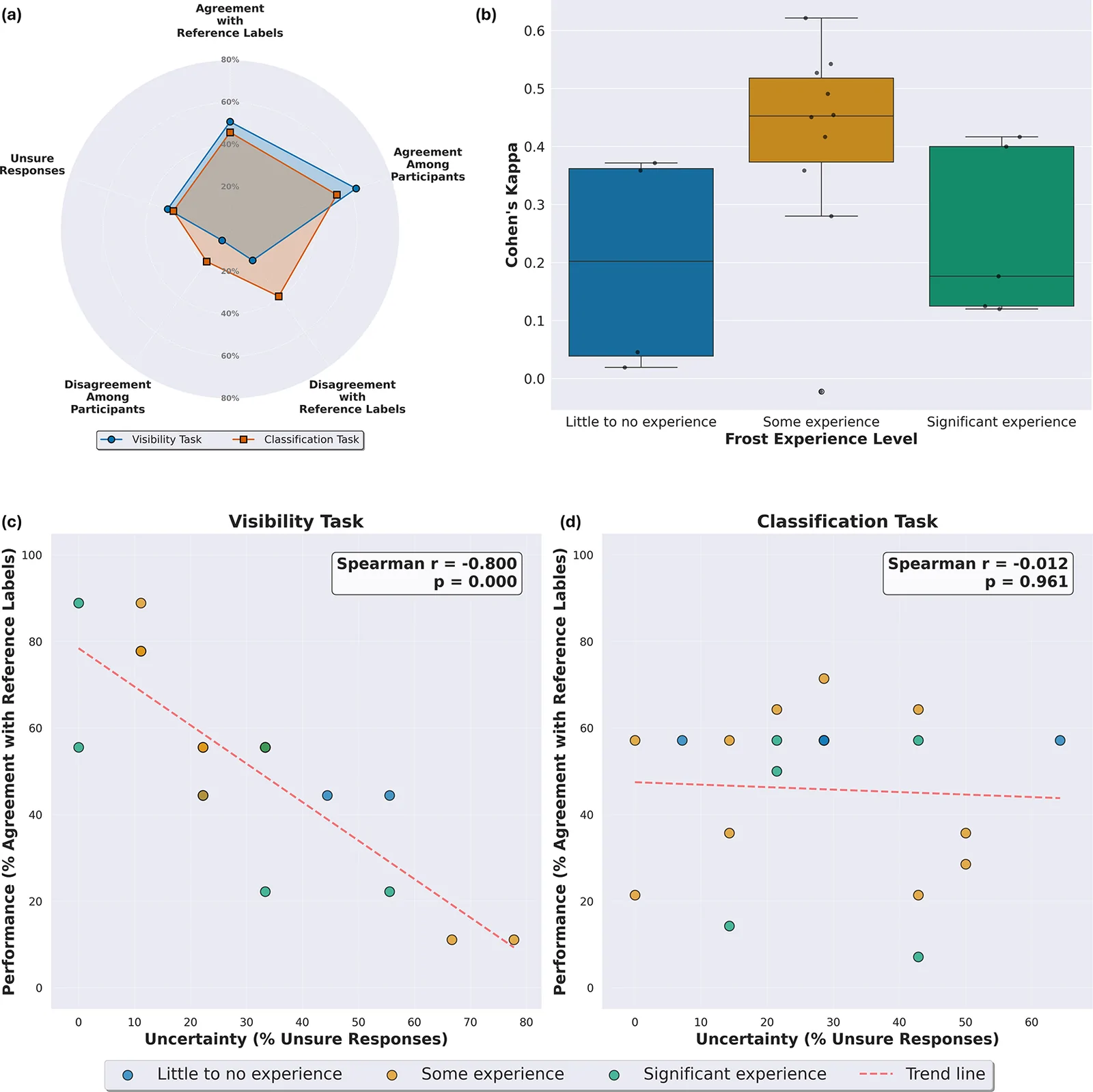
Summary of survey results evaluating expert reliability in frost identification and classification. (a) Comparison of response patterns between first screening (in the legend is indicated as visibility task) and classification tasks across five reliability metrics (0–80% scale): agreement with reference labels, inter-participant consensus, disagreement with reference labels, disagreement among participants, and unsure response frequency. (b) Distribution of inter-rater agreement (Cohen’s Kappa) against the ground truth across frost experience levels during classification tasks, showing an inverted U pattern where participants with moderate frost experience have higher consistency among participants than participants with little to no experience and significant experience with frost-action. (c) Relationship between uncertainty (% of unsure responses) and performance (% of agreement with ground truth) for the first-screening task, colored by frost-experience level. The red dashed line shows downward trend, indicating strong negative correlation (Spearman r = −0.800, p < 0.001). (d) Relationship between uncertainty and performance for the classification task, colored by frost-experience level. Flat red dashed line indicates no correlation (Spearman r = −0.012, p = 0.961).

Overall, agreement among experts is low in both tasks. Fleiss’s κ indicated only slight agreement (κ ≤ 0.20; McHugh (2012)) ( Table 1C). Figure 6a shows the broad spread of responses in both visibility and classification tasks relative to reference labels. The first-screening tasks produced roughly twice as many “Unsure” responses as the classification tasks, showing that experts find it harder to determine a feature that is present in the cropped and resized photomicrographs than to identify its class.

Individual performance varies widely. For the classification task, Cohen’s κ relative to reference labels range from near chance to moderate agreement ( Landis and Koch, 1977; McHugh, 2012), and percentage agreement per image ranges from 7 to 71%. In the first-screening tasks, percentage agreement ranges from 11 to 89%, but Cohen’s κ could not be calculated because all reference labels are identical (assuming that all features are visible; Supplementary Information 4, Section S4.7). Frost-specific experience does not follow linear patterns. Experts with “some experience” show the highest median Cohen’s κ (~ 0.45), while those with little to no experience (~ 0.20) and “significant experience” (~ 0.17) score lower with greater variability, producing an inverted U-shape pattern ( Figure 6b).

Experts select “Unsure” for approximately 25–33% of images on average, with large individual variation (Supplementary Information 4, Table S4.7.4). In the first-screening task, uncertainty is negatively correlated with performance (Spearman r = −0.800, p < 0.001; Figure 6c), while no correlation is observed for the classification task (Spearman r = −0.012, p = 0.961; Figure 6d).

### Models and experts show complementary strengths in frost classification

To compare models with expert performance, we evaluated how models’ predictions aligned with reference labels and with the 19 experts who classified the same images in the survey’s first screening and classification task. Extended results are provided in Supplementary Information 3 and 4.

On the nine first-screening images, models classified images confidently even when experts judged the images as insufficient for classification. Across the four images categorized as “Not visible” by majority vote, most models predicted an even split between frost and non-frost (2 each), with the exception of EfficientNetV2B0, which predicted frost for three of the four images. Mean confidence scores were higher for “Visible” images than “Not visible”, and confidence remained high than dropping to chance level, with BNN showing the clearest differentiation ( Table 2 and Figure 8). In the classification task, model agreement with reference labels varies significantly (Friedman x ^2^ = 33.4, p < 0.001; Table 1A). MobileNetV2 achieved the highest alignment with reference lables (Cohen’s κ = 0.536) and EfficientNetV2 performed well (κ = 0.490), with both significantly outperforming ResNet50.

**Table 2.** Mean confidence scores and predicted mean probability for each model on the nine-first screening images, categorized by expert majority vote. Confidence is computed as the absolute rescaled distance from the decision boundary. P mean is the mean predicted probability of frost across images in each category.

Model	Not visible		Visible	
	Confidence	P mean	Confidence	P mean
**Custom CNN**	0.722	0.616	0.954	0.733
**BNN**	0.516	0.707	0.863	0.754
**MobileNetV2**	0.557	0.637	0.852	0.741
**EfficientNetV2B0**	0.860	0.776	0.984	0.751
**ResNet50**	0.870	0.548	0.937	0.744

**Figure 7.**
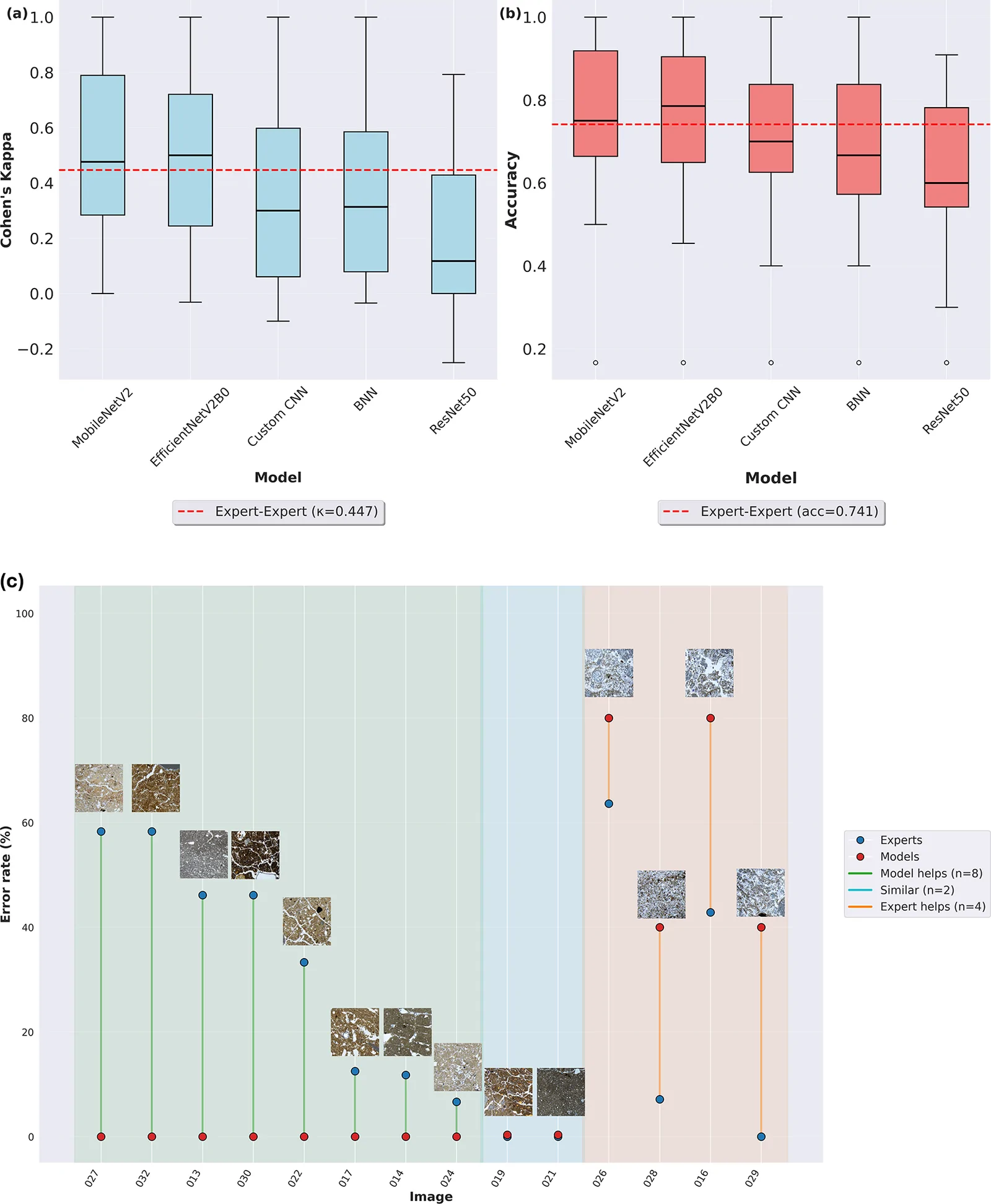
Expert-model agreement and image-level complementarity analysis. (a-b) Distribution of Cohen’s kappa (a) and accuracy (b) for each CNN model against 19 experts. Red dashed lines indicate the expert-expert baseline (κ = 0.447, accuracy = 0.741). MobileNetV2 achieves the highest alignment with experts, approaching the expert-expert baseline. (c) Comparison of expert error rates (blue dot) and model error rates (red dot) for each of the 14 classification images, with thumbnail previews. Lines connect expert and model error rates per image, colored by complementarity patterns: green lines indicate images where models outperform experts (n = 8), cyan lines indicate similar performance (n = 2), and orange lines indicate images where experts outperform models (n = 4). Background shading corresponds to these categories.

**Figure 8.**
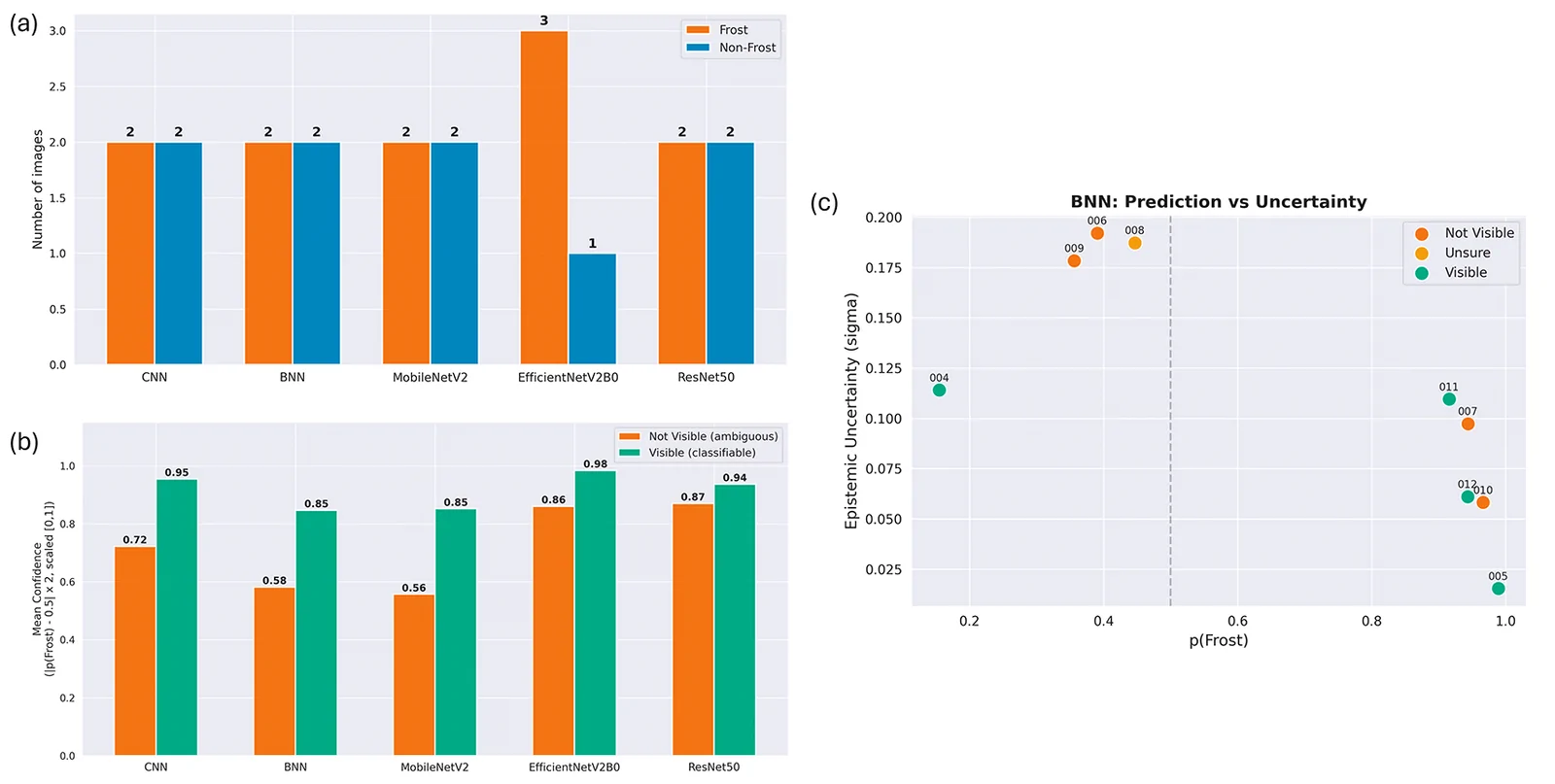
Model predictions, confidence, and epistemic uncertainty on the first-screening images. (a) Frost vs. non-frost predictions per model on the four images classified as “Not visible” by expert majority vote. (b) Mean confidence scores per model on “Not visible” and “Visible” image subset. Confidence scores are computed as rescaled distance from the decision boundary. (c) BNN predicted probability p(Frost) against epistemic uncertainty (σ, T = 20 Monte Carlo dropout passes) for all nine first-screening images, colored by expert visibility category. The dashed vertical line marks the 0.5 decision boundary between frost and non-frost classes.

Pairwise expert-model (95 pairs) and expert-expert (171 pairs) comparisons showed 70% and 74% accuracy respectively ( Figure 7a-b; Table 1B). Differences are not statistically significant for either κ (p = 0.224) or accuracy (p = 0.125), showing that CNN models achieve expert-level inter-rater reliability.

Agreement patterns differ within groups ( Table 1C). The five models show substantial consensus agreement (Fleiss’ κ = 0.676, >90% mean agreement), exceeding inter-rater agreement (Fleiss’ κ = 0.202, ~ 61% mean agreement; McHugh (2012)). When models are combined with experts (n = 24), Fleiss’ κ (0.212, fair agreement; ( Landis and Koch, 1977) approaches expert-only levels.

Image-level analysis reveals independent error patterns, where models and experts fail on different images, demonstrating complementary capabilities ( Figure 5c). Eight images are difficult for experts, and models help in classification; four images need expert verification in the models’ prediction, and in two models, experts’ and models’ classifications align. Finally, BNN uncertainty shows an inverse relationship with expert-perceived difficulty (Spearman ρ = −0.497, p = 0.071). Where experts expressed the highest uncertainty (~32-63% “Unsure” rate), BNN uncertainty remained low ( Figure 8c).

## Discussion

This study evaluates whether CNN-based classifications can identify frost features in archaeological thin sections with accuracy and reasoning comparable to practicing micromorphologists. We assessed model performance and interpretability, quantified expert variability, and directly compared models and expert classifications to determine whether computational and human approaches provide complementary capabilities. This framework enables us to evaluate not only classification accuracy but also decision mechanisms and the conditions under which CNNs can meaningfully support micromorphological practice.

We report four main findings: (1) CNN models achieve high classification performance, but best-performing off-the-shelf models rely on spurious correlations rather than diagnostic micromorphological features; (2) expert agreement on the same classification task is low, reflecting inherent interpretive challenges rather than methodological failure; (3) models and experts make independent errors on different images, indicating complementary strengths rather than redundant capabilities; and (4) model uncertainty does not align with expert-perceived difficulty, highlighting the need for expert oversight in hybrid workflows.

All models performed better on binary (frost vs. non-frost) than multiclass (blocky, granular, platy-lenticular) classification, and off-the-shelf models (MobileNetV2, EfficientNetV2B0, and ResNet50) performed better than those trained from scratch (Custom CNN, and BNN), a result that is consistent with the advantages of transfer learning for small-to-medium datasets reported in geological and petrographic imaging ( Cai et al., 2024; Dawson et al., 2023; Pires de Lima et al., 2020). Among three widely adopted CNN architectures, lightweight models (MobileNetV2 and EfficientNetV2B0) generalized more effectively that heavier architectures (ResNet50), likely because their simpler designs are less prone to overfitting on limited training data ( M. Romero et al., 2020b; Zhao et al., 2024). A similar pattern was also observed in carbonate rock classification where more complex CNN architectures performed better with bigger datasets ( Dawson et al., 2023). The performance drop from binary to multiclass classification reflects a fundamental challenge, and several factors contribute to this performance decline. First, the classification threshold in the binary is easier to learn, and multiclass classification threshold boundaries might be vague or overlap with another class ( Moral et al., 2022). Second, frost microstructures form a visual and functional continuum rather than discrete categories. Blocky, granular and platy-lenticular forms can be transitional, poorly developed or overlapping with other pedogenic processes through equifinality, for example granular microstructures from freeze-thaw cycles can resemble those produced from bioturbation ( Van Vliet-Lanoë, 1985). This overlap between categories contrasts with machine learning techniques that demand discrete categorical boundaries, creating an epistemological mismatch between natural variability and the fixed category labels required for classification ( Petrelli, 2024).

Furthermore, interpretability analysis showed that classification accuracy did not necessarily reflect the use of meaningful micromorphological features. Score-CAM maps showed that the highest-accuracy off-the-shelf models (MobileNetV2, EfficientNetV2B0, ResNet50) focused on scattered or central image regions unrelated to diagnostic criteria. This pattern is consistent with shortcut learning where deep networks exploit spurious regularities rather than focusing on the actual sediment features ( Adebayo et al., 2020; Geirhos et al., 2020; Rosenfeld et al., 2018). In our case, the spatial attention patterns observed in off-the-shelf models likely reflect biases inherited from ImageNet pretraining, where images are predominantly object-centric and models focus on the part of the image with the most variability ( Zhang et al., 2022). In ResNet50, the repeated activation of a central rectangular region across all photomicrographs exemplifies this center-of-the-photo bias. The additional quantitative Score-CAM analysis we conducted confirms that decisions in these off-the-shelf models are driven by compact regions of the image that only partially (or do not) overlap with micromorphological diagnostic sediment features, such as porosity and matrix arrangement, highlighting that high performance can coexist with non-diagnostic attention patterns.

In contrast, Custom CNN and BNN showed lower performance (varying from 86–95% in accuracy) but had clearer interpretability. Quantification of the pixel activation mass showed that the attention is more diffused, and Score-CAM overlays emphasized mostly on sediment matrix textures, frost-related porosity, and aggregate boundaries and shape, indicating that simpler architectures may force attention onto visual features that align with diagnostic criteria ( Lipton, 2017; Rudin, 2019). This interpretability-performance trade-off suggests that simpler architectures focus on diagnostic features but lack representational capacity to integrate them across the frost pattern for robust generalizations ( Raghu et al., 2019). At the same time, difference in feature extraction design across architectures (inverted residual blocks in MobileNetV2 ( Sandler et al., 2019), compound-scaled blocks in EfficientNetV2B0 ( Tan and Le, 2021), residual and squeeze and excitation blocks in ResNet50 ( He et al., 2015), and the more linear custom CNN and BNN) and the specific last convolutional layer used for Score-CAM introduce distinct inductive biases in which regions are treated as informative.

In addition, the crop-level splitting may contribute to the optimistic performance estimates through spatial autocorrelation between crops from the same photomicrograph. However, the robustness test on the entire photomicrographs, which is independent splitting strategy, reproduces the same architecture-dependent pattern: off-the-shelf models retain better classification performance while custom models show better interpretability alignment. This suggests that the core findings are not primarily by crop level autocorrelation.

Therefore, high classification performance alone is insufficient for evaluating models in micromorphological contexts, where feature identification must be grounded in diagnostic criteria. Interpretability analyses are then essential not merely to explain model behavior, but to verify that classifications reflect the same process-based reasoning that experts apply ( Ebert-Uphoff and Hilburn, 2020; Rudin, 2019). Similar interpretability paradoxes have been reported in geological petrography, where well-performing CNN’s focus on visually irrelevant or non-diagnostic regions ( Cai et al., 2024; Zheng et al., 2024). In other words, without explicit interpretability validation, deep learning models in micromorphology may be “right for the wrong reasons” and thus unreliable for process-based archaeological interpretations.

Interestingly, our survey results showed only a slight expert agreement both on ambiguous images and with reference labels. This reflects how micromorphological analysis typically unfolds: dynamically scanning thin sections while switching between cross-polarized and plane-polarized light, changing magnifications, rotating stages to observe crystal orientations, checking the overall geological context. In the survey, we effectively removed these workflows by providing static images at fixed lighting and resolution without a more general geological provenience context. Furthermore, many of the photomicrographs provided to the experts had weakly developed frost features or features that mimic those related to frost action (e.g., developed pedogenic pedality). Experts therefore lacked crucial information for confident classification, explaining low agreement, high uncertainty rates, and disagreement with the reference labels.

These constraints intersect with deeper challenges. Frost features can overlap with other pedogenic (planar voids and peds in vertisols) or biogenic (coalescent granular structures derived from bioturbation) processes, producing microstructures that can mimic those typically derived from frost action, particularly at the photomicrograph scale ( Stoops, 2021). This overlap is compounded by the continuum-to-category mismatch discussed above, which amplified the subjectivity in expert judgement (where to draw boundaries) and rigidity in model classification framework, which must assign a single category. Shahack-Gross (2016) showed similar low agreement among micromorphologists when looking at basic component and mineralogical identification, suggesting an interpretive variability in micromorphology analysis. Such subjectivity arises because micromorphological features vary in degree of development (from incipient to well-developed structures), diagnostic criteria involve development of quality judgments, and expert experience shapes recognition patterns.

This inherent subjectivity, however, also explains why model-experts complementarity is advantageous. The near-zero error correlation between models and experts indicates that machine versus human expert errors arise from distinct heuristics: where CNN models ask, “what does this look like?”; experts ask, “how did this form?”. These are different epistemic questions, meaning that CNNs are not simply faster versions of experts but different kinds of observers. Complementarity arises not from matching abilities but from non-overlapping strengths. Because models and experts succeed under different conditions, hybrid workflows are justified ( Dvijotham et al., 2023). Once validated, CNNs can rapidly screen large image sets, provide consistency unaffected by fatigue, and flag uncertain cases. Experts can then adjudicate ambiguous cases, provide contextual interpretation, and integrate into sedimentary and paleoenvironmental narratives; neither replaces the other ( Hemmer et al., 2025). This epistemic divergence is further evidenced by BNN uncertainty estimates, which were well calibrated for model predictions but inversely correlated with expert-perceived difficulty; where experts expressed uncertainty due to equifinality, missing context, or weak feature expression, the BNN remained confident because visual patterns matched training distributions. This confirms that Monte Carlo captures parameter uncertainty, not interpretive ambiguity ( Gal and Ghahramani, 2016). Expert oversight therefor remains essential for low-confidence cases, non-diagnostic attention patterns as revealed through the expert-models comparison and interpretability analysis, especially when specific images challenge all classifiers ( Bianchi et al., 2024; Steyvers et al., 2022).

However, several limitations constrain the generalizability of our findings. Our simplified classification scheme does not capture the full spectrum of frost action variation. The five CNN architectures evaluated here represent a subset of available deep learning approaches, and other architectures designed for spatial relationship capture may better accommodate the relational reasoning central to microfeatures. Reliance on PPL at single magnification eliminates information from XPL examination and multi-scale observation central to standard micromorphological practice.

While our dataset encompasses eleven Plio-Pleistocene sites, broader testing across diverse periglacial and non-periglacial contexts is needed to establish robust generalization. In particular, validation on fully independent datasets from sites not represented in the training data would be a critical next step for establishing model transferability across different sedimentary contexts, depositional environments, and imaging setups.

Future work should expand datasets across more sediment types and depositional environments, incorporate multiple imaging modalities, and explore more model architectures suited for capturing spatial relationships between microfeatures. A promising direction would be the development of multi-scale architectures that explicitly integrate both local patch-level learning, capturing the diagnostic criteria such as aggregate geometry and frost-related porosity, as well as photomicrograph-level context, enabling feature localization across scales. In this study, we took a preliminary step in this direction by evaluating model performance on both cropped patches and photomicrographs, demonstrating that patch level training captures diagnostic detail in the broader-photomicrograph spatial scale. Building on this, future work could develop models that combine both scales (or more) in a unified framework alongside with expert-annotated diagnostic regions as additional training signals. This would directly constrain models’ attention to micromorphologically meaningful features rather than spurious image regularities.

Beyond architectural and dataset considerations, future work will investigate methodological modifications to the training pipeline. As datasets increase in size and are more diverse, systematic comparisons of random and strict crop-level and photomicrograph-level splitting strategies would provide insights into the conditions in which each approach achieves the best balance between sample size, generalizability, and data independence. This comparison allows for establishing best practices for dataset preparation in micromorphological deep learning workflows.

Additionally, the low inter-expert agreement observed in our survey suggests that single label annotations may not fully capture the interpretive variability inherent to micromorphological classification. The survey itself is subject to several constraints that limit its broader generalizability. The sample size (n =19) and uneven distribution of self-reported experience levels with the majority reporting intermediate frost-specific experience, reflect the specialized and relatively small nature of practicing micromorphologists, aspects that dictated our deliberately descriptive and exploratory treatment of the survey results throughout this study. Our survey was also based on voluntary recruitment, which further introduces potential self-selection bias toward micromorphologists familiar with computational approaches or interested in machine learning integration. Together these limitations suggest that the survey findings are better understood as an exploratory baseline rather than a representative benchmark of micromorphological practice broadly. Future work should incorporate multi-annotator protocols that preserve disagreement as information rather than resolving it into single labels, thus enabling uncertainty-aware training strategies that reflect genuine diagnostic ambiguity ( Sheng and Zhang, 2019).

To facilitate the adoption and reproducibility of this framework, we provide a publicly accessible classification tool that implements the two-stage classification pipeline with interpretability maps, enabling researchers to obtain frost feature predictions for their own thin-section images with full transparency into model attention patterns (available at: https://huggingface.co/spaces/skkou/frost_classifier). However, the outputs should be treated as a first-pass suggestion and not as definitive micromorphological diagnoses and must be checked with established criteria and site context.

## Conclusions

This study evaluated whether deep learning models can identify and classify frost-related micromorphological features at an expert level while maintaining interpretability aligned with established diagnostic criteria. We trained five CNN models on photomicrographs from multiple archaeological sites and validated them against both interpretability analysis and an expert survey.

Three principal findings emerge. First, off-the-shelf models achieve better classification performance, but interpretability analysis revealed reliance on spurious correlations rather than diagnostic features, demonstrating that good performance alone is not sufficient for evaluating models in micromorphology. Second, expert agreement on the same task was low, confirming that inter-observer variability is intrinsic to frost feature identification rather than a limitation specific to computational approaches. Third, mode and expert errors were largely independent, indicating that CNNs and expert micromorphologists capture different and complementary aspects of frost feature recognition.

Together, these findings establish a proof-of-concept for model-expert collaboration that addresses persistent challenges in archaeological micromorphology: scaling comparative analysis beyond what the manual examination permits, improving reproducibility through consistent pattern recognition, and broadening analytical access for researchers without extensive frost-specific training. The proposed framework, combining interpretability validation, uncertainty quantification, multi-expert consensus labelling, and model-expert workflow, provides a transferable template for computational integration across disciplines facing similar challenges with subtle, overlapping features subject to inter-observer variability.

Expanding this framework to incorporate broader sedimentary contexts and multiple imaging modalities will be essential for moving towards computational micromorphological tools. An effective implementation requires integrated workflows, where models handle the first pass screening while experts provide targeted validation and contextual synthesis into behavioral narratives. This complementarity preserves interpretive integrity while enabling analyses at scales previously constrained by time and expertise availability.

## Ethics and consent

No ethical approval was needed for this study. The survey was completely anonymous, and we used no identifiable information on the survey participants.

## Data Availability

All data underlying this study are available from the Open Science Framework (OSF): OSF: Frost Action Classification. https://doi.org/10.17605/OSF.IO/TYDSM ( Kouki, 2026a). The project includes the following data:
• Image dataset used for classification • Image classification results • Survey results • Expert-CNN results • Supplementary tables and figures Data are available under the terms of the Creative Commons Attribution 4.0 International license (CC BY 4.0). Extended data are available from the Open Science Framework (OSF): https://doi.org/10.17605/OSF.IO/TYDSM ( Kouki, 2026a). The project contains the following extended data:
• Additional statistical analysis of model performance • Statistical analysis of expert survey data • Expert-CNN comparison analysis • Additional plots and tables • Guidelines for using the interactive classification tool Analysis code available from: https://github.com/SofiaKouki/Frost-Action-Classification.git Archived analysis code at time of publication: https://doi.org/10.17605/OSF.IO/TYDSM ( Kouki, 2026b). License: CC0 1.0. Software available from: https://huggingface.co/spaces/skkou/frost_classifier Source code available from: https://github.com/SofiaKouki/Frost-Action-Classification.git Archived source code at time of publication: https://doi.org/10.17605/OSF.IO/TYDSM ( Kouki, 2026b). License: CC0 1.0.
