# Supplementary material for: Automated detection of freeze-thaw signatures in archaeological sediments using deep learning
Source: Open Res Eur. 2026 Aug 4;6:144. Originally published 2026 May 2. [Version 2] doi: 10.12688/openreseurope.23697.2 (PMC13560803; doi:10.12688/openreseurope.23697.2)
Supplement: Peer review history

# Reviewer report: Reviewer response for version 2

**DOI:** 10.21956/openreseurope.26759.r78563

**Published:** 2026-08-22

**Authors:** Mathias Bellat

**Affiliations:** 1 University of Tübingen, Tübingen, Germany

All the concerns expressed earlier have been addressed. I therefore would recommend indexing.

If any, are all the source data and materials underlying the results available?

Yes

Is the work original in terms of material and argument?

Yes

Is the work clearly and cogently presented?

Yes

Is the argument persuasive and supported by evidence?

Yes

Does the research article contribute to the cultural, historical, social understanding of the field?

Yes

Does it sufficiently engage with relevant methodologies and secondary literature on the topic?

Yes

Reviewer Expertise:

Archaeological sciences (machine learning); Southwestern Asia Neolithic archaeology; Landscape archaeology

I confirm that I have read this submission and believe that I have an appropriate level of expertise to confirm that it is of an acceptable scientific standard.

# Reviewer report: Reviewer response for version 2

**DOI:** 10.21956/openreseurope.26759.r78562

**Published:** 2026-08-19

**Authors:** Jerome G. Gacu

**Affiliations:** 1 Kyoto University, Kyoto, Japan

All comments were resolved.

If any, are all the source data and materials underlying the results available?

No source data required

Is the work original in terms of material and argument?

Partly

Is the work clearly and cogently presented?

Partly

Is the argument persuasive and supported by evidence?

Partly

Does the research article contribute to the cultural, historical, social understanding of the field?

Partly

Does it sufficiently engage with relevant methodologies and secondary literature on the topic?

Yes

Reviewer Expertise:

Sediment management, water resources engineering

I confirm that I have read this submission and believe that I have an appropriate level of expertise to confirm that it is of an acceptable scientific standard.

# Reviewer report: Reviewer response for version 1

**DOI:** 10.21956/openreseurope.25655.r73575

**Published:** 2026-06-15

**Authors:** Alexandra Karamitrou

**Affiliations:** 1 University of Southampton, Southampton, UK

**Structure**

 Although the supplementary material contains substantial detail, the manuscript itself needs additional explanation and visual support to be fully accessible to non‑expert readers. Consider integrating key figures and clarifying complex sections within the main text.

**Clarity**

 - In the *Machine Learning Workflow* section, please provide additional detail on each method used, including relevant citations. This will help readers understand the rationale behind your choices and how each component contributes to the workflow.

 - The *Experts and CNN Models Alignment* section would be clearer if the results were summarized in a table. This would allow you to remove the dense explanatory text at the end of the section and present the findings more effectively.

**Figures**

**-**The *Materials and Methods* section would benefit from including example photomicrographs, as well as a map showing the locations of all sampled materials. These additions would greatly improve clarity for readers unfamiliar with the dataset.

 - Figure S.4.2.1 from the supplementary material should be incorporated into the main manuscript. It provides a clear visual representation of the workflow, which is difficult to follow when described only in text.

 - While the supplementary material is extensive, the manuscript itself would benefit from relocating some of these figures into the main text to support non‑expert readers.

**Technical Corrections**

**-**“All the images we acquired are in RGB format, with a resolution of 2880 x 2048-pixel resolution and saved as uncompressed.tif files.” 

 Correct to:

 “All the images we acquired are in RGB format, with a resolution of 2880 x 2048-pixel and saved as uncompressed.tif files.”

**Supplementary material**

 I can verify that the code runs correctly and that all necessary documents are accessible through the provided links.

If any, are all the source data and materials underlying the results available?

Yes

Is the work original in terms of material and argument?

Yes

Is the work clearly and cogently presented?

Partly

Is the argument persuasive and supported by evidence?

Yes

Does the research article contribute to the cultural, historical, social understanding of the field?

Yes

Does it sufficiently engage with relevant methodologies and secondary literature on the topic?

Yes

Reviewer Expertise:

My area of research is in the use of artificial intelligence and remote sensing data (or images) to detect and map different types of archaeological targets or targets of archaeological interest.

I confirm that I have read this submission and believe that I have an appropriate level of expertise to confirm that it is of an acceptable scientific standard, however I have significant reservations, as outlined above.

# Response

**Published:** 2026-07-23

**Authors:** Sofia Kouki

We sincerely thank the reviewer for their constructive and insightful feedback, as well as for the time and effort dedicated to reviewing our manuscript. Below, we address each comment point-by-point.   Structure Although the supplementary material contains substantial detail, the manuscript itself needs additional explanation and visual support to be fully accessible to non‑expert readers. Consider integrating key figures and clarifying complex sections within the main text. We have now added additional figures and tables from the Supplementary Information to the manuscript.

 Specifically, the new additions include the workflow image (Figure 2), the confusion matrices of the misclassifications (now as Figure 4a), and additional images of the model-expert comparison (now as Figure 8). Clarity - In the Machine Learning Workflow section, please provide additional detail on each method used, including relevant citations. This will help readers understand the rationale behind your choices and how each component contributes to the workflow.

 We revised parts of the Machine Learning workflow and provided the rationale behind each step and how each one of them contributes to the workflow (lines: 136-163, 155-158, and 162-168) - The Experts and CNN Models Alignment section would be clearer if the results were summarized in a table. This would allow you to remove the dense explanatory text at the end of the section and present the findings more effectively. We revised the Expert and CNN model Alignment section, and we added a table and a figure (now Table 2 and Figure 8) to facilitate non-expert readers.

 Figures -The Materials and Methods section would benefit from including example photomicrographs, as well as a map showing the locations of all sampled materials. These additions would greatly improve clarity for readers unfamiliar with the dataset. We included a figure that contains a map with the sites used for this study and an example of photomicrographs from the locations (now Figure 1).

 We also created an interactive map of the sites that contain representative photomicrographs from the sites, and we included it in the OSF repository so it can be publicly accessible (10.17605/OSF.IO/TYDSM). - Figure S.4.2.1 from the supplementary material should be incorporated into the main manuscript. It provides a clear visual representation of the workflow, which is difficult to follow when described only in text. Figure S4.2.1 from the Supplementary Information is now incorporated into the main text as Figure 2. - While the supplementary material is extensive, the manuscript itself would benefit from relocating some of these figures into the main text to support non‑expert readers.

 We included additional figures and tables from the Supplementary Information in the main text to support non-expert readers (now as Figure 2 and 8 and Table 2). Technical Corrections -“All the images we acquired are in RGB format, with a resolution of 2880 x 2048-pixel resolution and saved as uncompressed.tif files.” Correct to: “All the images we acquired are in RGB format, with a resolution of 2880 x 2048-pixel and saved as uncompressed.tif files.” Corrected. Supplementary material I can verify that the code runs correctly and that all necessary documents are accessible through the provided links. We thank the reviewer for this verification.

# Reviewer report: Reviewer response for version 1

**DOI:** 10.21956/openreseurope.25655.r75439

**Published:** 2026-06-11

**Authors:** Jerome G. Gacu

**Affiliations:** 1 Kyoto University, Kyoto, Japan

1. Add a methodology framework figure in the main manuscript showing image acquisition, preprocessing, data splitting, CNN training, Score-CAM analysis, expert survey, and model validation workflow.

 2. Redesign Figure 1 using a common x-axis and shared legend. Increase subplot size to improve readability and utilize page space more efficiently.

 3. In Figure 2, add arrows, circles, or labels to identify the specific sediment features causing model misclassification. The current examples are difficult to interpret without annotations.

 4. Figure 3, use a single shared color bar and legend for all Score-CAM outputs. Increase image size and annotate representative regions showing spurious attention and diagnostically relevant features.

 5. Perform a strict photomicrograph-level train-validation-test split and compare results with the current crop-level split. Report performance differences in the main manuscript.

 6. Add a table summarizing final hyperparameters for each model, including learning rate, batch size, dropout rate, optimizer, epochs, and regularization settings.

 7. If possible, validate the best-performing model using an independent dataset from a different archaeological site to demonstrate model generalizability.

 8. Simplify Figure 5c or divide it into two figures. The current figure is visually crowded and difficult to interpret.

 9. Include 95% confidence intervals for all reported performance metrics (Accuracy, Precision, Recall, and F1-score).

 10. Add a discussion on the limitations of the expert survey, particularly the small sample size and uneven distribution of expertise levels.

If any, are all the source data and materials underlying the results available?

No source data required

Is the work original in terms of material and argument?

Partly

Is the work clearly and cogently presented?

Partly

Is the argument persuasive and supported by evidence?

Partly

Does the research article contribute to the cultural, historical, social understanding of the field?

Partly

Does it sufficiently engage with relevant methodologies and secondary literature on the topic?

Yes

Reviewer Expertise:

Sediment management, water resources engineering

I confirm that I have read this submission and believe that I have an appropriate level of expertise to confirm that it is of an acceptable scientific standard, however I have significant reservations, as outlined above.

# Response

**Published:** 2026-07-23

**Authors:** Sofia Kouki

We would like to thank the reviewer for reading our manuscript and providing meaningful comments that improved our draft. We appreciate the time and effort dedicated to reviewing our work. Below, we address each comment in turn.  

 1. Add a methodology framework figure in the main manuscript showing image acquisition, preprocessing, data splitting, CNN training, Score-CAM analysis, expert survey, and model validation workflow. We added a workflow image to the main draft, now as Figure 2. 

 2. Redesign Figure 1 using a common x-axis and shared legend. Increase subplot size to improve readability and utilize page space more efficiently. We have redesigned the figure (now as Figure 1) in response to this comment. Specifically, all subplots now have a shared x-axis across both rows, legends are consolidated into a single shared legend, and we increased the size to improve readability as suggested.

 3. In Figure 2, add arrows, circles, or labels to identify the specific sediment features causing model misclassification. The current examples are difficult to interpret without annotations. We revised the image by adding arrows and lines, showing better the features that cause models’ misclassifications.

 4. Figure 3, use a single shared color bar and legend for all Score-CAM outputs. Increase image size and annotate representative regions showing spurious attention and diagnostically relevant features. We revised the figure as suggested; it is now Figure 5 in the manuscript.

 5. Perform a strict photomicrograph-level train-validation-test split and compare results with the current crop-level split. Report performance differences in the main manuscript. We thank the reviewer for raising this point regarding dataset splitting, and we assume that this comment is raised because of the potential risk of performance inflation due to crop-level dependency. We agree that in microscopic imaging, executing data splits at the independent image or slide level is another way to test absolute model generalizability across sediments. We have added a paragraph (lines: 609-619) to discuss these data partitioning dynamics, and we acknowledge that the spatial autocorrelation bias is not fully neutralized. However, we respectfully argue that for this specific scope, sample size constraints, and primary objectives of this study, our current crop-level splitting strategy is methodologically justified and robust. We have chosen not to repartition and retrain the models at the strict photomicrograph level for the following reasons: Targeted crop curation: In standard computer vision applications, crop-level data leakage occurs when an image is sliced into redundant, overlapping grid tiles that capture the exact same uniform background signal. In our workflow, crops were not arbitrary spatial subdivisions. Instead, they were deliberately curated to retain only regions with diagnostic features, while discarding non-diagnostic elements (e.g., limestone fragments and slide edges). Because of this strict semantic filter, crops originating from the same photomicrograph do not represent redundant samples of a singular background texture; rather, each retained crop captures a distinct diagnostic observation. This makes the crop-level more appropriate analytical unit for our task. Contextual diversity and sample-size constraints: Our models were tasked with generalizing across diverse archaeological sites with different depositional processes. Because diagnostic freeze-thaw features can be localized and do not distribute uniformly across the entire slide, a strict photomicrograph level split across the entire dataset would have restricted the network’s exposure to minority feature variation per site. The crop-level split allowed us to maximize the model’s learning capabilities and ensure stable convergence across the range of heterogeneous contexts. To verify the validity of our choice, we conducted an independent, whole photomicrograph-level robustness test (discussed in the manuscript in lines 194-201 and 376-378). As detailed in Supplementary Information 4, Section 4.6, this independent evaluation confirmed that the comparative architectural behaviors and interpretability trends reported remain robust. Nonetheless, we added in the Discussion an explicit mention for future integration of this slide-level split as the underlying dataset expands. 

 6. Add a table summarizing final hyperparameters for each model, including learning rate, batch size, dropout rate, optimizer, epochs, and regularization settings. A comprehensive summary of final hyperparameters for all five models is already included in Supplementary Information 4, Section S2.4, Table 4.2.2. In the manuscript there is an explicit reference to this table in the Materials and Methods section (Materials and Methods, Machine Learning Workflow, paragraph 4). We believe that retaining the hyperparameter table in the supplementary material is appropriate given the level of technical detail involved, which would otherwise interrupt the flow of the method section, while remaining fully accessible to readers who wish to reproduce or build upon our workflow.

 7. If possible, validate the best-performing model using an independent dataset from a different archaeological site to demonstrate model generalizability. We fully agree with the reviewer that validation on an independent dataset from different archaeological sites would strengthen the generalizability claims of the current models, and we recognize this as a priority for future work. Unfortunately, at this stage we do not have access to additional independently collected thin section material from sites outside our current dataset that would constitute a truly independent validation set. However, we would like to draw the reviewer’s attention to several steps we did toward generalizability within the constraints of our current dataset: (1) our dataset already spans 11 archaeological sites with diverse periglacial and non-periglacial depositional environments; (2) our robustness test on full photomicrographs provides an additional evaluation that is independent of the crop-level splitting strategy; and (3) our held-out test set was kept entirely separate from training and validation throughout. To this end, we have added an explicit statement to the limitations and future work section of the discussion acknowledging independent dataset validation as a critical next step, alongside a broader sedimentary context and depositional environments that we discussed already (lines: 603-606).

 8. Simplify Figure 5c or divide it into two figures. The current figure is visually crowded and difficult to interpret. We thank the reviewer for this suggestion. We acknowledge that in the previous version of the manuscript, Figure 5c (now Figure 7c) conveys a lot of information; however, this plot is designed to simultaneously convey per-image error rates for both experts and models, their directional relationship, and complementary patterns across all 14 classification images. We believe this format is the most efficient way to present these four dimensions of information in a single panel without losing both modeling and micromorphological details. The thumbnail photomicrographs are an intentional design choice that allows readers to directly connect classification difficulty patterns to the images.

 9. Include 95% confidence intervals for all reported performance metrics (Accuracy, Precision, Recall, and F1-score). We have now included the 95% confidence intervals for all reported metrics in the figure (now as Figure 3). These changes were implemented alongside the figure redesign requested in Comment 2. We also updated the image legend and caption to include the CI. 10. Add a discussion on the limitations of the expert survey, particularly the small sample size and uneven distribution of expertise levels. We have expanded the discussion of the expert survey constraints to the limitations and future work section of the discussion (lines 627-641), where it sits alongside the other methodological limitations of the study. We believe that this placement is more consistent with the overall structure of the discussion while ensuring the limitations are clearly acknowledged as the reviewer proposed.

# Reviewer report: Reviewer response for version 1

**DOI:** 10.21956/openreseurope.25655.r73580

**Published:** 2026-06-02

**Authors:** Mathias Bellat

**Affiliations:** 1 University of Tübingen, Tübingen, Germany

**Relevance of the manuscript to the archaeological field:**

 This manuscript presents a new methodology for discerning frozen features in thin sections using an image recognition algorithm. By comparing different deep learning and other types of models with experts' assessments, it distinguishes differences between frozen and non-frozen sections, and, for the frozen section, three types of classes. Results show better performance of two deep learning models compared to expert-expert agreement, while the models' attention is not drawn to the distinctive criteria.

**Abstract and Keywords:**

Background section: "Identifying these features through thin section micromorphology is well established" I don‘t see the meaning of "well established"; consider replacing it with something else, like "these features are possible to identify under a microscope ".

Methods section: "(first presence, then feature type)" Please consider adding the notion of *multiclass labelling* to distinguish these two approaches from the abstract onward.

**Article**

Major comments:

 I think the switch from §1.4 to §1.5 in the introduction is a little bit too straightforward. You should briefly introduce the computational approaches before explaining WHY they are particularly suited for frozen action. In my opinion, your construction should be :

 Frozen action description (§1.3) -> difficulty of identification of these features (§1.4 without last sentence) -> computational approaches may fill this "Gap" (reframe §1.5) -> explain principles and limitations of the computational approaches (§1.6 - §1.8).

 This work should not take long; all the elements are already there and well explained, it is just a matter of logical organisation of the arguments, which might lose a non-specialist reader.

*Site selection and data preparation:*

 Did you also have several experts attest to the identification of thin-section features, or was it done by only one researcher?

Minor comments:

*Introduction:*

 "microenvironments", while I understand the idea, I do not entirely agree. Features revealing wetter conditions (vesicle, riozome) can be linked with larger climatic conditions, therefore not "micro". I think environments alone is sufficient and more adapted to all possible conditions.

 Regarding the interpretability of the model, while your approach is entirely justified and correct, you might consider adding a few words on the SHAP value analysis, which also allows interpretability for image classification (https://shap.readthedocs.io/en/latest/image_examples.html).

*Material and methods:*

 "In our case, a 5x magnification provides a balance between the feature resolution". While you justify the choice of this magnification above, are you not afraid of missing many feature observations? Rizhoms or desiccation can only be seen at higher magnification most of the time. Same for the silt and clay structure.

 With ImageNet weights models, did you use a specific pre-trained version of the algorithms? In which case, you can cite the affiliated papers or GitHub repositories to enhance reproducibility.

 "We evaluated all models' performance using accuracy, precision, recall,  F1-scores, and confusion matrices, as well as visualising the misclassified images per mode". Could you provide the equations for each metric? Integrate the S4.1 info somehow into the appendix or in the text.

 "Fleiss’ kappa, individual expert-reference labels alignment using Cohen’s kappa " *Idem* for these metrics

 What is the demography of the experts? In the supplementary material, I could not find any information on the academic background (researcher, field archaeologist, technicians) or geographical location (country of affiliation) of the experts.

*Workflow and interactive classification tool:*

 I just wanted to highlight the quality of the tool the authors produced. This truly enhanced the accessibility of the research to non-developers.

*Results*:

 "Overall, all five models (MobileNetV2, EfficientNetV2B0, ResNet50,  Custom CNN, and BNN) perform well on both binary (frost vs. non-frost)  and multiclass (blocky, granular, and platy-lenticular) tasks in both  5-fold cross-validation and the test set. " Please do not use the element's **good result**; this does not make sense if you are not comparing your results to other models. Use the range of your score instead.

 "Agreement between experts and model approaches the level of agreement observed between experts." I don‘t understand this sentence.

*Discussion:*

 Could you think of a way – for future applications -  to constrain a deep learning model to focus on diagnostic elements rather than just a patch?

**Language**

 "Israel, Levant", while I do not agree with the current politics of Israel, I think you should remove the " Levant " mention for a site which is not considered part of the state of Palestine. If you have a specific issue with this mention, please contact the editors, as I cannot assess this kind of decision as a reviewer.

 "uncompressed.tif " lacks a space between the word and the later dot.

**Graphics, images and tables**

 Consider adding a map showing all site locations and a distinct sign for the site experiencing frozen or non-frozen conditions.

 Legend of Fig.4, the "(0–80% scale)" word uses a double score instead of a single score.

 Please consider adding the supplementary figure Figure S.4.2.1 to the core text.

 I could not verify the code due to my platform system (ArchLinux) 

**Bibliography**

 The authors have provided all necessary references regarding the topic. I would suggest additional references, however, as they might bring elements regarding the presented subject

**Additional references:**

 Zickel, M., Gröbner, M., Röpke, A., & Kehl, M. (2024). MiGIS: Micromorphological soil and sediment thin section analysis using an open-source GIS and machine learning approach. *E&G Quaternary Science Journal*, *73*(1), 69–93. https://doi.org/10.5194/egqsj-73-69-2024

If any, are all the source data and materials underlying the results available?

Yes

Is the work original in terms of material and argument?

Yes

Is the work clearly and cogently presented?

Yes

Is the argument persuasive and supported by evidence?

Yes

Does the research article contribute to the cultural, historical, social understanding of the field?

Yes

Does it sufficiently engage with relevant methodologies and secondary literature on the topic?

Yes

Reviewer Expertise:

Archaeological sciences (machine learning); Southwestern Asia Neolithic archaeology; Landscape archaeology

I confirm that I have read this submission and believe that I have an appropriate level of expertise to confirm that it is of an acceptable scientific standard, however I have significant reservations, as outlined above.

## References

[1] : MiGIS: micromorphological soil and sediment thin section analysis using an open-source GIS and machine learning approach. *E&G Quaternary Science Journal* .2024;73(1) : 10.5194/egqsj-73-69-202469-93 10.5194/egqsj-73-69-2024

# Response

**Published:** 2026-07-23

**Authors:** Sofia Kouki

We would like to thank the reviewer for reading our manuscript and the constructive comments. We appreciate the time and effort dedicated to evaluating our work. Below, we address each comment in turn.

**Relevance of the manuscript to the archaeological field:** This manuscript presents a new methodology for discerning frozen features in thin sections using an image recognition algorithm. By comparing different deep learning and other types of models with experts' assessments, it distinguishes differences between frozen and non-frozen sections, and, for the frozen section, three types of classes. Results show better performance of two deep learning models compared to expert-expert agreement, while the models' attention is not drawn to the distinctive criteria.****

**Abstract and Keywords:** Background section: "Identifying these features through thin section micromorphology is well established" I don‘t see the meaning of "well established"; consider replacing it with something else, like "these features are possible to identify under a microscope ". This sentence was changed to read: “Frost-associated features can be identified through soil micromorphology techniques using well-defined diagnostic criteria”.  We chose to retain the term “well-defined” because it reflects not only the ability to examine soils and sediments under a microscope but also the long-established application of soil micromorphology to the study of frost-affected sites. Moreover, this technique preserves the original geometry of the deposits, allowing the investigation of pore geometries that would otherwise be impossible to identify.   Methods section: "(first presence, then feature type)" Please consider adding the notion of multiclass labelling to distinguish these two approaches from the abstract onward. We changed the sentence to "...implementing a two-stage classification approach by detecting first the presence of those features (binary classification) and then the feature type (multiclass classification)," see Abstract Method section.

**Article** **Major comments:** I think the switch from §1.4 to §1.5 in the introduction is a little bit too straightforward. You should briefly introduce the computational approaches before explaining WHY they are particularly suited for frozen action. In my opinion, your construction should be: Frozen action description (§1.3) -> difficulty of identification of these features (§1.4 without last sentence) -> computational approaches may fill this "Gap" (reframe §1.5) -> explain principles and limitations of the computational approaches (§1.6 - §1.8). This work should not take long; all the elements are already there and well explained, it is just a matter of logical organization of the arguments, which might lose a non-specialist reader. We have reorganized the sections as requested (lines 41-48).****

**Site selection and data preparation:** Did you also have several experts attest to the identification of thin-section features, or was it done by only one researcher? The rationale and the labeling procedure are described in Supplementary Information 4 (Section 4.2, Data labeling), and we expanded this section to contain extensive details about this topic.

**Minor comments:** **Introduction:** "microenvironments", while I understand the idea, I do not entirely agree. Features revealing wetter conditions (vesicle, riozome) can be linked with larger climatic conditions, therefore not "micro". I think environments alone is sufficient and more adapted to all possible conditions. We agree with the reviewer, and we have changed the word from "microenvironments" to “environments” as suggested (line 9).  Regarding the interpretability of the model, while your approach is entirely justified and correct, you might consider adding a few words on the SHAP value analysis, which also allows interpretability for image classification ( https://shap.readthedocs.io/en/latest/image_examples.html). We thank the reviewer for this suggestion. We have revised the relevant sentence in the introduction to broaden the interpretability methods with an explicit acknowledgement of SHAP (lines: 55-58)  

**Material and methods:**  "In our case, a 5x magnification provides a balance between the feature resolution". While you justify the choice of this magnification above, are you not afraid of missing many feature observations? Rizhoms or desiccation can only be seen at higher magnification most of the time. Same for the silt and clay structure. We would like to clarify that rhizomorphs and larger desiccation features fall outside the scope of this study’s classification framework, which specifically targets aggregate-scale frost microstructures (granular, blocky, and platy-lenticular). These microstructures are defined by aggregate geometry, porosity distribution, and spatial relationships between features, which are all optimally captured at 5x magnification. Higher magnifications would resolve fine fabric details but would simultaneously lose the spatial contexts necessary to distinguish between the three target microstructure classes. The 5x magnification was therefore not a compromise, but a deliberate methodological choice driven by the classification objectives. With ImageNet weights models, did you use a specific pre-trained version of the algorithms? In which case, you can cite the affiliated papers or GitHub repositories to enhance reproducibility. The ImageNet pre-trained weights for all three off-the-shelf architectures (MobileNetV2, EfficientNetV2B0, and ResNet50) were loaded through the standard Keras applications API, with versions determined by Tensorflow environment reported in the manuscript. This information is also contained in the requirements.txt file on GitHub where the code is publicly available (https://github.com/SofiaKouki/Frost-Action-Classification.git).

 We added a clarifying sentence in lines 295-297 to showcase the pre-trained model with their original papers cited.  "We evaluated all models' performance using accuracy, precision, recall,  F1-scores, and confusion matrices, as well as visualising the misclassified images per mode". Could you provide the equations for each metric? Integrate the S4.1 info somehow into the appendix or in the text.  "Fleiss’ kappa, individual expert-reference labels alignment using Cohen’s kappa " Idem for these metrics We have now expanded the existing glossary table in Supplementary Information 4 (Table S4.1.1) to include formal equations for all performance metrics and statistical measures used in this study, alongside their definitions and references.

 We believe this provides a cleaner and more accessible reference for readers without adding unnecessary complexity to the text. We point to the Supplementary Information in the main text for further information on the metrics for the readers to look for (line: 280). What is the demography of the experts? In the supplementary material, I could not find any information on the academic background (researcher, field archaeologist, technicians) or geographical location (country of affiliation) of the experts. This is a valid point, as we only collected self-reported frost experience levels as the most directly relevant proxy for experience, and we acknowledged the limited demographic characterization as a design limitation in the manuscript. However, there are two main reasons why we chose not to collect demographic information on participants’ academic background or geographical affiliation.

 First, given the small sample size (n = 19) and the specialized nature of the micromorphological community, collecting such details would risk identifying individual participants, compromising the ethical integrity of the survey. Second, and more fundamentally, demographic variables play no role in our statistical analysis. The expertise level we report is used descriptively rather than as the basis for inferential conclusions and adding further demographic stratification to a sample of 19 participants would not yield statistically meaningful results.  Still, we agree with the reviewer that more in-depth surveys would be of interest to assess variance in micromorphologists' identification of frost action as well as of other processes. This is an important avenue for future research but lies beyond the scope of the present study.  

**Workflow and interactive classification tool:** I just wanted to highlight the quality of the tool the authors produced. This truly enhanced the accessibility of the research to non-developers. We thank the reviewer for the supportive comment. 

**Results**: "Overall, all five models (MobileNetV2, EfficientNetV2B0, ResNet50,  Custom CNN, and BNN) perform well on both binary (frost vs. non-frost)  and multiclass (blocky, granular, and platy-lenticular) tasks in both  5-fold cross-validation and the test set. " Please do not use the element's good result; this does not make sense if you are not comparing your results to other models. Use the range of your score instead. We have replaced the evaluative descriptors with specific performance ranges for binary and multiclass classification tasks, respectively (lines: 290-293) "Agreement between experts and model approaches the level of agreement observed between experts." I don‘t understand this sentence. We have removed the unclear sentence and retained the more explicit reporting of pairwise agreement values, sample sizes, and statistical significance in the following sentence, which more clearly conveys that models achieve inter-rater reliability comparable to that of human experts (lines: 446-449).

**Discussion**: Could you think of a way – for future applications -  to constrain a deep learning model to focus on diagnostic elements rather than just a patch? We agree with the reviewer that this is a meaningful future application. To express that, we have now added a full paragraph in this direction in the limitations and future work section of the Discussion (lines: 665-671).

**Language** "Israel, Levant", while I do not agree with the current politics of Israel, I think you should remove the " Levant " mention for a site which is not considered part of the state of Palestine. If you have a specific issue with this mention, please contact the editors, as I cannot assess this kind of decision as a reviewer. In the spirit of consistency, we have removed the "Levant," and we kept the countries' names. "uncompressed.tif " lacks a space between the word and the later dot. Corrected.

**Graphics, images and tables** Consider adding a map showing all site locations and a distinct sign for the site experiencing frozen or non-frozen conditions. We have added a map showing the site locations to the main draft, now as Figure 1. Legend of Fig.4, the "(0–80% scale)" word uses a double score instead of a single score. Corrected. Please consider adding the supplementary figure Figure S.4.2.1 to the core text. We have added the workflow image to the main draft, now as Figure 2.  I could not verify the code due to my platform system (ArchLinux). The code is publicly accessible through the GitHub repository (https://github.com/SofiaKouki/Frost-Action-Classification.git) and archived on OFS (10.17605/OSF.IO/TYDSM), along with the saved models. The entire workflow was implemented in Google Collaboratory, a cloud-based platform that runs independently of the user’s local operating system and is accessible through any standard web browser.  On the GitHub page, there are also guidelines on how to implement the pipeline without depending on platform constraints.

**Bibliography** The authors have provided all necessary references regarding the topic. I would suggest additional references, however, as they might bring elements regarding the presented subject  Additional references: Zickel, M., Gröbner, M., Röpke, A., & Kehl, M. (2024). MiGIS: Micromorphological soil and sediment thin section analysis using an open-source GIS and machine learning approach. E&G Quaternary Science Journal, 73(1), 69–93. https://doi.org/10.5194/egqsj-73-69-2024 Zickel et al. (2024) differs methodologically from our approach, since the authors rely on traditional machine learning rather than deep learning, and operating on whole thin section scans. Nevertheless, in the interest of providing a comprehensive overview of existing computational approaches in thin section analysis through soil micromorphology, we have added Zickel et al. (2024) to the introduction along with the rest of the studies, noting its distinct scope and methodology (lines: 69-72).
